# Biophysical and Structural Features of αβT‐Cell Receptor Mechanosensing: A Paradigmatic Shift in Understanding T‐Cell Activation

**DOI:** 10.1111/imr.13432

**Published:** 2024-12-29

**Authors:** Robert J. Mallis, Kristine N. Brazin, Jonathan S. Duke‐Cohan, Aoi Akitsu, Hanna M. Stephens, Ana C. Chang‐Gonzalez, Daniel J. Masi, Evan H. Kirkpatrick, Elizabeth L. Holliday, Yinnian Feng, Katarzyna J. Zienkiewicz, Jonathan J. Lee, Vincenzo Cinella, Kaveri I. Uberoy, Kemin Tan, Gerhard Wagner, Haribabu Arthanari, Wonmuk Hwang, Matthew J. Lang, Ellis L. Reinherz

**Affiliations:** ^1^ Laboratory of Immunobiology Dana‐Farber Cancer Institute Boston Massachusetts USA; ^2^ Department of Medical Oncology Dana‐Farber Cancer Institute Boston Massachusetts USA; ^3^ Department of Medicine Harvard Medical School Boston Massachusetts USA; ^4^ Department of Dermatology Harvard Medical School Boston Massachusetts USA; ^5^ Department of Chemical and Biomolecular Engineering Vanderbilt University Nashville Tennessee USA; ^6^ Department of Biomedical Engineering Texas A&M University College Station Texas USA; ^7^ Structural Biology Center, X‐Ray Science Division, Advanced Photon Source Argonne National Laboratory Lemont Illinois USA; ^8^ Department of Biological Chemistry and Molecular Pharmacology Harvard Medical School Boston Massachusetts USA; ^9^ Department of Cancer Biology Dana‐Farber Cancer Institute Boston Massachusetts USA; ^10^ Department of Materials Science and Engineering Texas A&M University College Station Texas USA; ^11^ Department of Physics and Astronomy Texas A&M University College Station Texas USA; ^12^ Center for AI and Natural Sciences Korea Institute for Advanced Study Seoul Republic of Korea; ^13^ Department of Molecular Physiology and Biophysics Vanderbilt University School of Medicine Nashville Tennessee USA

**Keywords:** cell signaling, mechanosensing, molecular dynamics (MD), optical tweezers (OT), preTCR, single molecule (SM), T cell, T‐cell receptor (TCR)

## Abstract

αβT cells protect vertebrates against many diseases, optimizing surveillance using mechanical force to distinguish between pathophysiologic cellular alterations and normal self‐constituents. The multi‐subunit αβT‐cell receptor (TCR) operates outside of thermal equilibrium, harvesting energy via physical forces generated by T‐cell motility and actin‐myosin machinery. When a peptide‐bound major histocompatibility complex molecule (pMHC) on an antigen presenting cell is ligated, the αβTCR on the T cell leverages force to form a catch bond, prolonging bond lifetime, and enhancing antigen discrimination. Under load, the αβTCR undergoes reversible structural transitions involving partial unfolding of its clonotypic immunoglobulin‐like (Ig) domains and coupled rearrangements of associated CD3 subunits and structural elements. We postulate that transitions provide critical energy to initiate the signaling cascade via induction of αβTCR quaternary structural rearrangements, associated membrane perturbations, exposure of CD3 ITAMs to phosphorylation by non‐receptor tyrosine kinases, and phase separation of signaling molecules. Understanding force‐mediated signaling by the αβTCR clarifies long‐standing questions regarding αβTCR antigen recognition, specificity and affinity, providing a basis for continued investigation. Future directions include examining atomistic mechanisms of αβTCR signal initiation, performance quality, tissue compliance adaptability, and T‐cell memory fate. The mechanotransduction paradigm will foster improved rational design of T‐cell based vaccines, CAR‐Ts, and adoptive therapies.

## 1 Introduction

### 1.1 Discovery and Structural Elucidation of the αβ T‐Cell Receptor

The αβTCR complex has been characterized during the past 40+ years in considerable detail since its initial identification using monoclonal antibodies and biochemical methods, subsequent molecular cloning, and structural definitions [1, 2, 3, 4, 5, 6, 7, 8, 9, 10]. Yet the precise mechanism of activation upon weak affinity binding by a peptide‐major histocompatibility complex ligand (pMHC) has remained enigmatic, particularly when one considers that an effective αβTCR must find and respond to as little as a single foreign pMHC in the context of tens of thousands of unrelated self pMHCs [11]. Furthermore, the topology of pMHC recognition by the TCRαβ clonotypic component of the holoreceptor has similarly been known for nearly 30 years [12, 13, 14] with hundreds of TCRαβ structures, their pMHC ligands, or complexes of TCRαβ and pMHC determined in subsequent years, without clear clues to the origins of distinct signaling potencies revealed from those static structures. Any effort at understanding signal progression had been hindered by the absence of a definitive description of the αβTCR holocomplex, which has only recently been defined, first by cryoEM in detergent micelles [15, 16], and then in more physiologic phospholipid nanodiscs [17] as described below. Other structural techniques, such as NMR [18, 19] or fluorescence‐based structural studies [20] have provided hints at conformational flexibility, but without conclusive proof of direct involvement in signal transduction pathways. In short, despite great effort and progress, the precise mechanism that drives a recognition signal from the T‐cell surface through the membrane to initiate and sustain the signaling cascade has not been revealed, although many explanations have been proposed [21]. This lack of a clear mechanistic definition impedes understanding the role of the αβTCR in fighting human disease, especially with the growing emphasis of utilizing T cells in immuno‐oncology [22] and adjacent biomedical applications [23].

### 1.2 Mechanotransduction as an Operating Principle of αβTCR Activation

αβTCR mechanosensing is a transformative paradigm that begins to bridge the gap in understanding αβTCR activation, providing new clues to the critical determinants of αβTCR fitness. The initial idea that mechanical force was the previously unaccounted factor came in studies exploring T‐cell activation. First, it was posited that the signal would more readily transmit from the TCRαβ into the adjacent CD3 subunits due to the rigidified central structural elements of the latter, as described below [24, 25]. It was later demonstrated that intact T‐cell adhesion and cytoskeleton were necessary for low pMHC copy number activation [26] and, separately, that bead‐bound pMHC would only activate T cells when force was applied in a direction parallel to the cell membrane [27]. Within this latter study it was suggested that such force‐responsiveness would be actuated or accompanied by catch bonds [27, 28, 29], a prediction borne out in studies interrogating intact T cells with a biomembrane force probe (BFP) [30] and then using optical tweezers (OT) on TCRαβ heterodimers in isolation or as intact holoreceptors on T‐cell surfaces [31].

The directionality of the applied force and force‐mediated triggering at limiting pMHC copy numbers was later refined and shown to be a result of coupling of the αβTCR to the actin cytoskeleton [32] cementing force as the central driver for sensitive αβTCR signaling. Involvement of the actin cytoskeleton during initial stages of αβTCR activation (prior to immune synapse formation) has also been observed in cell‐level experiments [33, 34]. Catch bond behavior was accompanied by reversible structural transitions within the TCRαβ ectodomain [31] and was found to be operative within the preTCR as well [35], which shares a common β subunit and CD3 components with the αβTCR. In contrast, catch bond formation is not a feature of γδTCRs [36] that possess a unique γδ clonotypic heterodimer while sharing CD3 substituents. These observations highlight that mechanotransduction is important enough to necessitate its operation during the earliest committed stage of the αβ lineage, and that αβT cells have a specialized requirement for mechanotransduction. Below we discuss each aspect of mechanotransduction in greater detail, including the specificity gating engendered by catch bonds, the perceived role of the reversible structural transitions, the implications of the loading direction of force and its transmission pathways through the αβTCR holoreceptor complex.

## 2 αβTCR Function From a Structural and Kinetic Perspective

### 2.1 A Structural Description of αβTCR‐pMHC Binding Events

**FIGURE 1:**
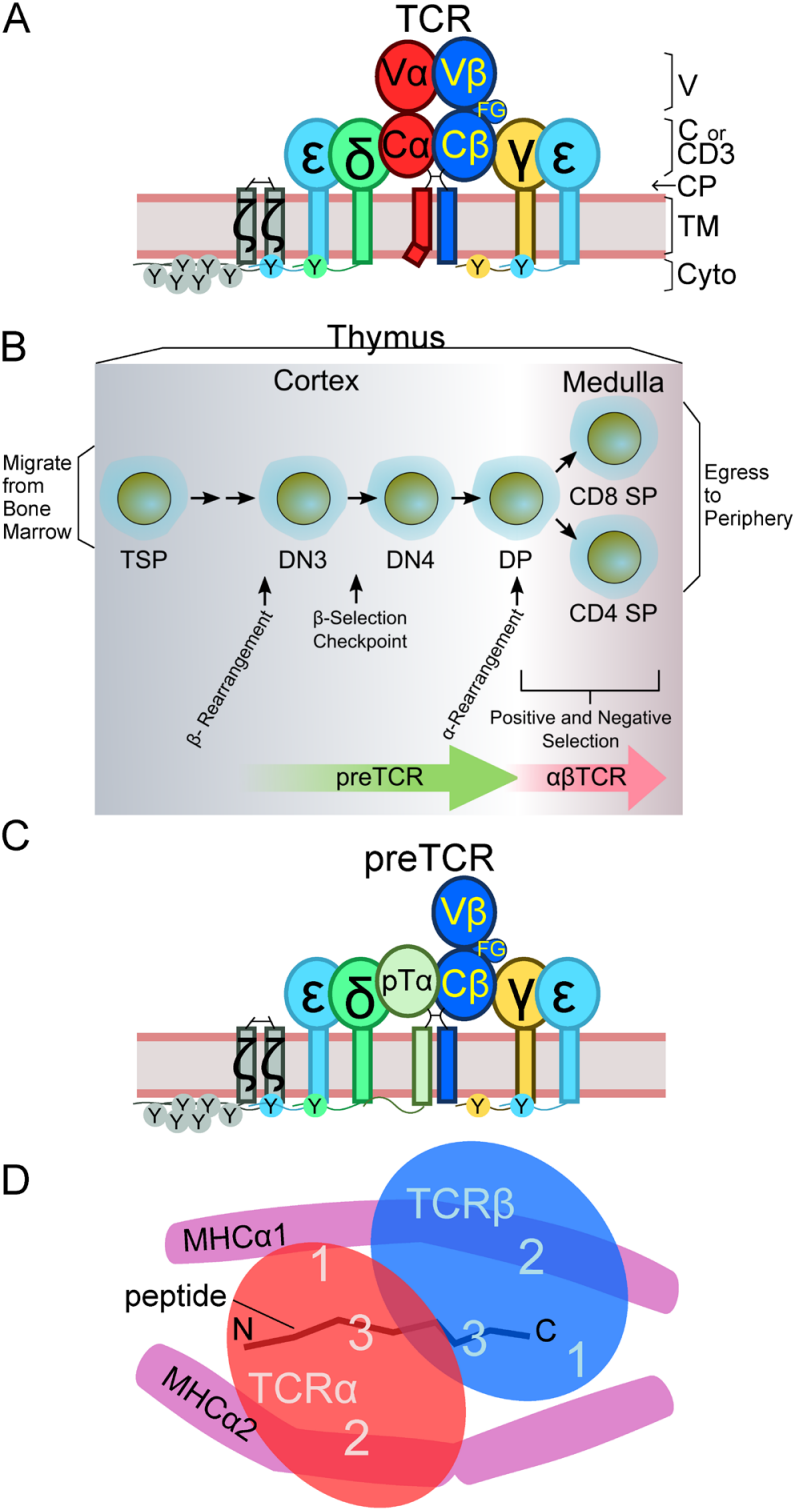
Organization, development and canonical ligand recognition of the αβTCR. (A) The αβTCR is a cell surface transmembrane receptor composed of eight subunits including the ligand binding TCRαβ heterodimer, signaling CD3 heterodimers εγ and εδ and CD3 homodimer ζζ. ITAM motifs including dual Tyr residues (denoted by a circled Y) are indicated in proper stoichiometry. The Cβ FG loop is labeled. Terminology for the αβTCR components from N to C terminus (top to bottom): Variable (V) domains; Constant (C) domains or CD3 ectodomains (CD3); Connecting Peptide region (CP); Transmembrane region (TM); Cytoplasmic Tail including ITAMs (Cyto). (B) Thymic selection occurs as hematopoietic progenitors migrate to the thymus, are committed to the T‐cell lineage (Thymus seeding progenitors; TSP) and undergo β‐rearrangement, β‐selection, α‐rearrangement and then positive and negative selection to mature into CD4 or CD8 single positive (SP) thymocytes prior to emigrating from the thymus to the peripheral tissues as naïve T cells. Immature SPs are omitted for clarity. Panel adapted from [37]. (C) The preTCR shares most components with the αβTCR except for substitution of pTα for TCRα. Annotation as in panel A. (D) Schematic footprint of TCRαβ interacting with pMHC ligand. View is from the TCR side where pMHC is behind the TCR. MHCI α‐helices from α1 and α2 subunits are shown. The CDR1‐3 ligand recognition loops' approximate interaction positions are shown with numbers within the red or blue ellipse for positioning of TCR Vα or Vβ domains. The peptide N and C termini are indicated. Positions are derived from the crystal structure of the N15αβ‐VSV8/K^b^ complex [38].

The αβTCR comprises a clonotypic (i.e., clone‐specific) TCRαβ transmembrane heterodimer non‐covalently associated with three invariant CD3 dimers, CD3εγ, CD3εδ, and CD3ζζ (Figure 1A). Ligand binding is governed by the αβ heterodimer, since the CD3 components lack pMHC binding capacity, with CD3ζζ entirely devoid of a folded ectodomain. On T cells, the CD4 or CD8 coreceptors directly interact with MHC Class II and I, (MHCII and MHCI) respectively, but with no peptide specificity [39, 40, 41], facilitated by TCRαβ binding to pMHC. Coupled to CD4 or CD8 is the Src family kinase Lck, which phosphorylates ITAMs in the cytoplasmic tails of CD3 subunits, to initiate the intracellular signaling cascade [42, 43, 44]. Mature T cells develop from hematopoietic precursors which migrate to the thymus, commit to T‐lineage development, and undergo successive somatic recombination processes to generate, initially, complete TCRβ‐ and then α‐encoding gene segments, thus progressing through a preTCR stage prior to the assembly of the mature αβTCR [reviewed in [45, 46]; Figure 1B]. This is notable in that the preTCR utilizes an invariant pTα chain in place of the α subunit of the mature αβTCR (Figure 1C). However, the β subunit of preTCR is precisely the same rearranged β chain that is utilized later for antigen recognition by an αβT‐cell clone which has matured, exits the thymus, and confronts its cognate antigen in the periphery (Figure 1B,C).

The binding of TCRαβ itself with pMHC has been dissected in detail elsewhere [8] and will be described here briefly. In general, the TCRαβ binds pMHC such that the β subunit, and CDR3β in particular, interacts with the C‐terminal half of the peptide, with CDR1β and 2β commonly interacting with the MHC itself. CDR3α interacts more towards the center to N‐terminal half of the peptide and, again, CDR1α and 2α mainly interact with the MHC (Figure 1D). Remarkably, most (two‐thirds to three quarters) of the TCRαβ recognition surface originates from self‐MHC [7]. The binding orientation of the TCRαβ is typically diagonal in relation to the long axis of the MHC‐bound peptide. This orientation allows the TCRαβ to recognize both of the α1 and α2 helices of the MHC that form the peptide binding groove. There is significant variation in the relative contribution of each TCRαβ subunit to the interface, however, as well as the orientation of the TCRαβ relative to the long axis of the peptide, that is, “docking angle”. This docking angle is generally restricted to less than 90° [38], which may originate from a germline bias for postulated recognition motifs [47, 48] and/or a fundamental geometric requirement for signal initiation [49, 50]. Despite this tremendous body of work, the initiation of signaling was not evident within static TCRαβ‐pMHC structures aside from the requisite geometry of the binding event itself.

**FIGURE 2:**
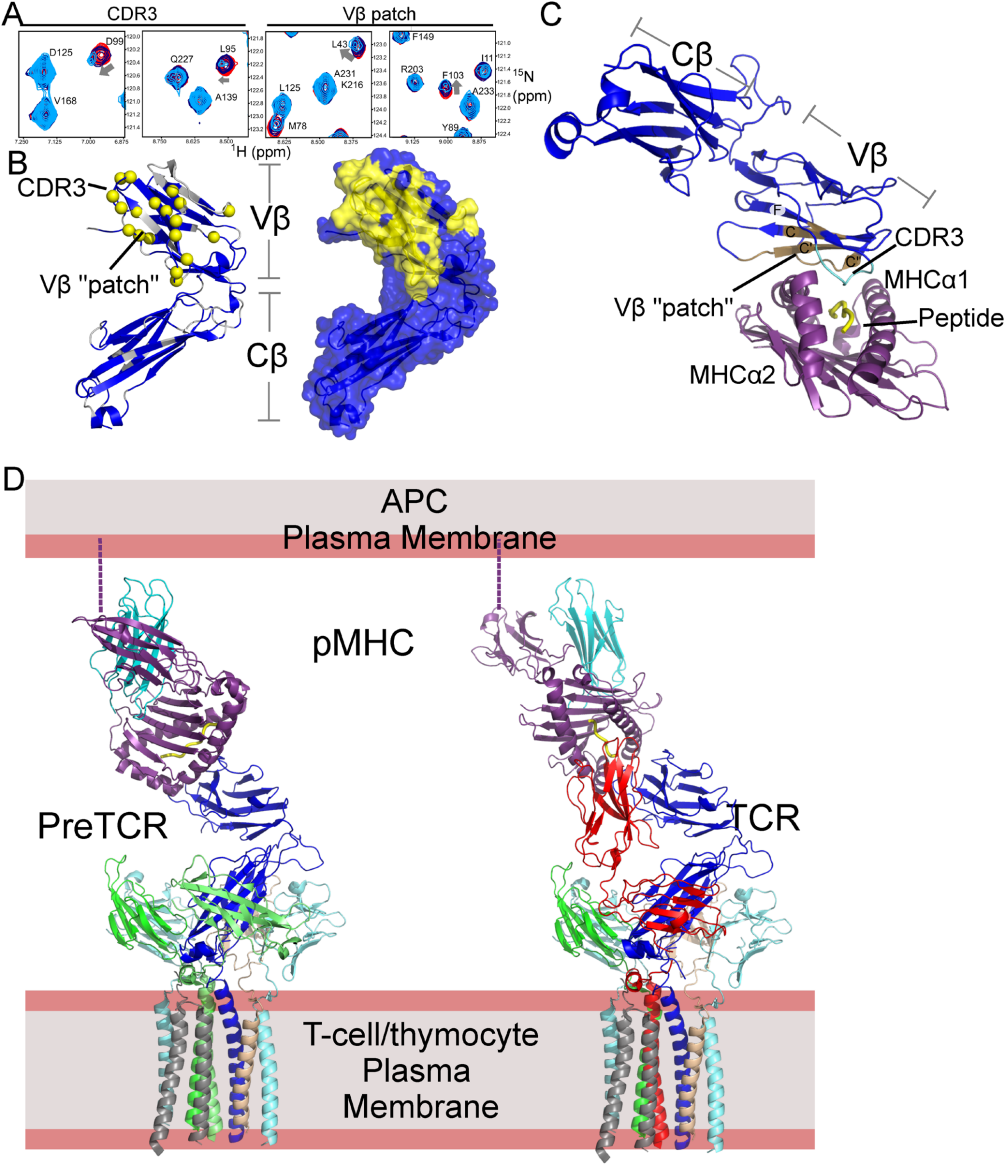
The preTCR recognizes pMHC ligand via a combined recognition surface utilizing CDR3 and the Vβ patch region with similarities and differences from TCR‐pMHC ligation. (A) Chemical shift perturbation data showing changes in ^1^H‐^15^N TROSY‐HSQC spectra with addition of VSV8/K^b^ to N15β used as a model for the preTCR ligand binding. Peaks are from N15β alone (red), with 200 (dark blue) and 500 μM (light blue) pMHC ligand added. (B) x‐ray crystallographic models of N15β with residues interacting with ligand highlighted. Left is a cartoon structure with a sphere indicating each interacting residue; Right is the corresponding surface model. Panels A‐B adapted from [51]. (C) Crystal structure of preTCR‐pMHC model N15β‐VSV8/K^b^ highlighting the interaction of CDR3 (cyan) with the C‐terminal residues of the peptide (yellow) and the Vβ framework residues including the Vβ patch (C″‐C′‐C‐F face; tan) interacting with MHC residues, particularly with the MHC α2 helix. (PDB code 6WL2). (D) Models of preTCR‐pMHC (left) and αβTCR‐pMHC (right) using crystal structures of N15β‐VSV8/K^b^ [53], N15αβ‐VSV8/K^b^ [38], and preTCR [55] overlaid on the cryoEM structure of the αβTCR [15]. Subunit coloring is as in Figure 1A,C. Heavy chain of K^b^ is purple, β2m is cyan and peptide is yellow. Despite the local differences in interaction angles, the longitudinal distance remains essentially the same, as does the incident angle with the antigen presenting cell (APC) plasma membrane assuming an upright TCR/preTCR. A dotted line depicts the C‐terminus of the MHC heavy chain connecting to the plasma membrane. The single‐span TM of MHC class I structure has not been determined to date. All structures rendered with PyMol [56].

Notably, the preTCR interacts with pMHC in a manner akin to, but with significant differences from the TCRαβ‐pMHC canonical interaction [51, 52, 53, 54]; (Figure 2A,B). These interfaces were first defined via solution NMR experiments with N15 preTCR‐VSV8(seq:RGYVYQGL)/K^b^, detailing canonical chemical shift changes along with site specific loss of resonance peak intensity at the binding interface [Figure 2A,B; [51]]. Loss of intensity is particularly noteworthy, as it suggests a slow to intermediate exchange rate on the order of hundreds of milliseconds to seconds between the bound and unbound states. This finding is surprising given the relatively weak binding, with a dissociation constant (K_D_) of 0.4 mM as measured by solution NMR [51]. The non‐canonical interface between the N15 preTCR and VSV8/K^b^ has been confirmed using isotopically labeled MHC in NMR studies, by another preTCR, and with orthogonal techniques including chemical crosslinking and X‐ray crystallography, providing additional details [51, 52, 53, 54]. The TCRβ subunit within the preTCR interacts with the C‐terminal residues of the peptide via its CDR3, while the body of the Vβ including the face of the C″‐C′‐C‐F strands, the “Vβ patch”, interacts with the α2 helix of the MHC [Figure 2B,C; [51, 52, 53]]. Aligning the preTCR‐pMHC complex with the αβTCR cryoEM structure [15] results in a putative intercellular interaction model with similar overall length and global approach to that of αβTCR‐pMHC (Figure 2D). However, CDR1 and 2 interactions have not been observed with preTCRβ‐pMHC, indicative of differences [53]. Moreover, there may be localized differences that could impact signaling by biasing force responses within the common CD3 subunits (Figures 1C and 2D). That said, there are some commonalities between the two systems including a clear force response for the preTCR with a critical force similar to that of the TCRαβ but with notable differences in transition rates (see below) and specificity [35, 51].

### 2.2 Responses of Binding Under Force: Force‐Lifetime Relationships and Conformational Changes

**FIGURE 3:**
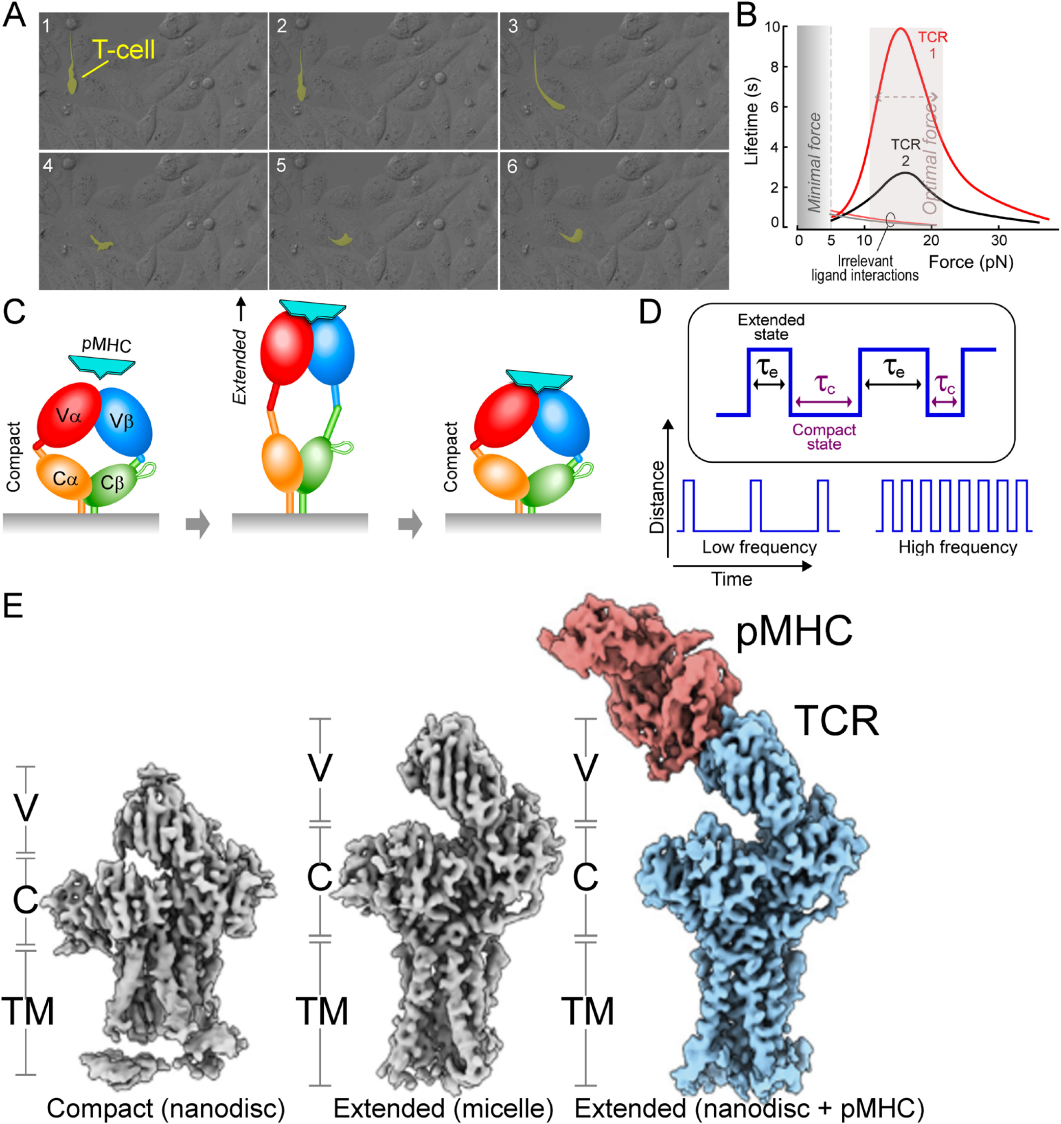
T‐cell motility engenders mechanical forces to modulate TCR ligand binding strength and reversibly deform the TCR to activate T‐cell signaling. (A) Serial images of a T cell scanning a stationary layer of epithelia as APCs in search of cognate antigen [57]. A T cell is highlighted in yellow to show approximate position and shape changes over time (frames 1 through 6 span approximately 1 s). This and another movie are available as Video S1 and S2. (B) Schematic bond‐lifetime vs. force curves showing two catch bond profiles of two different TCRs interacting with the same cognate ligand (red and black) and the slip bond profiles of an irrelevant ligand. Relevant force regimes are highlighted. (C) TCR reversible transitioning model. Cycles of extension and contraction under load as observed in OT experiments are shown for ectodomains of T‐cell surface αβTCR and pMHC. CD3 subunits, TM regions and cytoplasmic tails are not shown for simplicity. (D) Schematic SM traces contrasting low‐ and high‐frequency transitions. (B–D) adapted from [58]. (E) Variability in the conformation of the αβTCR with addition of pMHC imaged with cryoEM. Left: Unliganded, compact state obtained in nanodisc. Middle: Unliganded, extended state obtained in detergent micelle. Right: pMHC liganded, extended state obtained in nanodisc. Approximate boundaries are indicated: V, TCRαβ Variable region; C, TCRαβ Constant region and CD3 ectodomains; TM, TCRαβ and CD3 TM regions. On the right panel, αβTCR and pMHC are colored for clarity. Structural image adapted from [17].

Movement of T cells and/or antigen presenting cells (APCs) during immune surveillance generates mechanical force linked to T cell‐APC recognition. In the examples shown in Figure 3A and Videos S1 and S2, the motile T cells are moving over a stationary epithelium in search of pMHC [57]. The subsequent recognition process exerts forces in the 10–20 pN range [59] that αβTCRs exploit for optimal ligand screening as evidenced by the forces engendering ligand‐specific catch bonds [30, 31, 32, 60, 61] and (illustrated in Figure 3B). These forces accelerate unbinding for irrelevant or self‐peptides. For peptides that are a high‐quality match with a given αβTCR, the bonds survive and these forces trigger structural transitions in the TCRαβ resulting in the TCRs adopting extended conformations under force. As the interactions are fine‐tuned for optimal force, these ligated receptors can undergo repeated cycles of extended and compact states. These cycles last for multiple seconds and even minutes in vitro and may last even longer in vivo [31, 60] (Figure 3C,D). Cycling through squat‐extended‐squat conformations occurs at up to 40 Hz transition frequency with about 10 nm in amplitude accommodated between the T cell and APC. For simplicity, only the TCRαβ heterodimer is visualized in Figure 3C.

Recent cryoEM results reveal the unligated αβTCR holoreceptor captured in a compact configuration in nanodiscs whereas it is in an extended and rigidified conformation upon binding pMHC [17]; (Figure 3E). The cryoEM‐captured extension may prime the complex to enter the loaded state and assist with the compact and extended state cycling. Importantly, the same extended conformation can be replicated by isolating the full receptor in digitonin or digitonin‐like micelles by cryoEM [15, 17]. This implies a profound effect of the native‐like membrane environment on TM conformation and on the overall structure as noted by Notti et al. [17]. Additionally, interactions between membrane lipid and the CD3 ectodomain were also found to be substantial in those cryoEM images. The pMHC ligated conformation without applied force is presumably one of multiple conformational states occurring after load application. The transfer of information, collectively, can be understood by studying not only spatial but also dynamic relationships within the holocomplex.

The structures of the CD3 ectodomains [25, 49] provide clues to the mechanisms of signal transmission by virtue of the dimeric ectodomain interdigitating interfaces and juxtaposing parallel β sheets with the rigidified membrane proximal CXXC motifs of each heterodimeric subunit [62]. These membrane proximal domains were found to mediate inter‐subunit interactions as shown for the TCRα membrane proximal connecting peptide (CP) with that of CD3δ via NMR [63] and confirmed by cryoEM [15]. While the mode of interaction for each CD3 is different, the cryoEM data shows that the CPs partially govern interactions between the CD3 heterodimers and the TCRαβ subunits. Intriguingly, the interaction between the CP of TCRα and CD3ζζ appears substantial and may explain the early dissociation of CD3ζζ with activation of the αβTCR [15, 16, 17, 63]. The TMs themselves each have distinctive features likely related to signaling function, as exemplified by the TCRα TM which harbors an intramembrane conserved charge as well as a kinked structure, both of which govern their signaling potential [63]. Although there are many proposals regarding the potential charge pairings within the αβTCR complex TM regions [64, 65, 66, 67, 68], it remains unclear how much these TMs associate as stable structures. It is known that CD3ζζ forms a homodimer within the membrane [69], and that there are commonalities in the cryoEM structures [15, 16, 17], but the dynamics of these TM associations need to be further elucidated beyond static structures [63]. There are also indications that the membrane composition is important for the TM structures and association, as cholesterol has been found within the TM bundle in the cryoEM structures [15, 16, 17, 70]. In isolation, the CD3 ectodomains appear to associate very weakly with the TCRαβ ectodomains and likely require the CPs and TMs for proper CD3 orientation within the complex. From the recent αβTCR cryoEM studies in nanodiscs [17] one can discern the potential for dynamic regulation of the αβTCR wherein it is unambiguously shown that the full αβTCR adopts distinct conformations within the lipid bilayer depending on the presence of pMHC ligand (Figure 3E). Without pMHC, TMs are in a splayed state and straighten and elongate upon pMHC binding to TCRαβ while the CD3 ectodomains shift relative to the TCRαβ subunits. Additionally, the CPs transition from a compacted to a less compacted state, accompanied by conformational changes. The fact that the micellar structures can mimic the pMHC bound structure [15, 17] suggests a low energy barrier to activation for these structural alterations. The cytoplasmic tail (CT) regions of the CD3 components are thought to be largely unstructured, but the ITAM regions appear to associate with the inner leaflet of the plasma membrane in a charge‐dependent and possibly Ca^2+^ potentiated manner [71, 72, 73, 74, 75]. As discussed below, membrane association of the ITAM‐bearing regions suggests a mechanism for force‐mediated activation.

### 2.3 Techniques Enabling Biophysical Characterization of the αβTCR Mechanosensor

**FIGURE 4:**
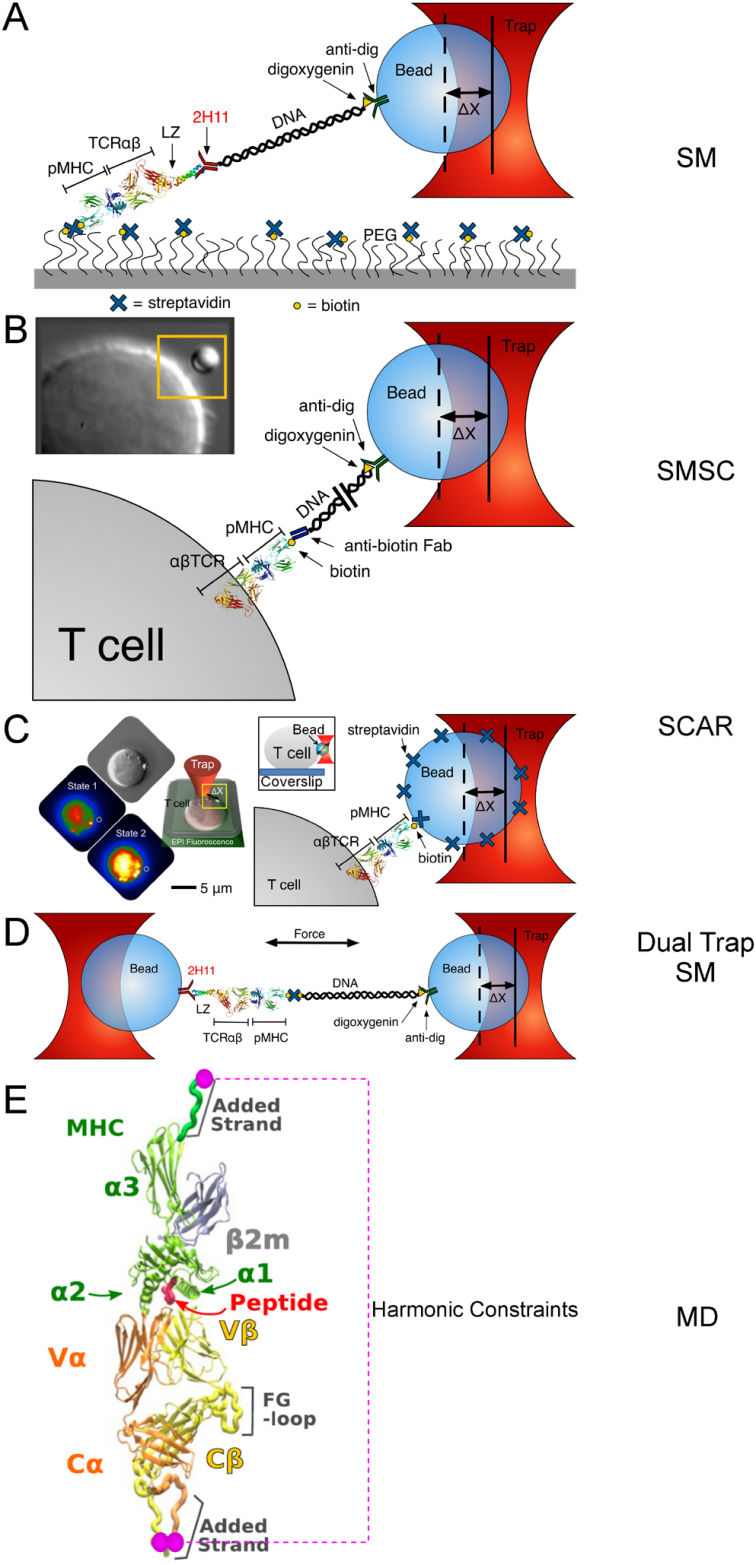
Biophysical investigation of TCR mechanosensor function. Each panel illustrates the experimental setup of the indicated assay. (A) Single molecule (SM). (B) Single molecule‐Single Cell (SMSC) with inset showing a bead approaching the T cell. Panels A‐B adapted from [31]. (C) Single Cell Activation Requirement (SCAR). Adapted from [32]. (D) Dual Trap Single Molecule (DTSM). Adapted from [60]. (E) Molecular dynamics (MD) simulation. Loads are applied via the harmonic restraining potential imposed on the C_α_ atoms of the terminal residues. Added strands allow transverse fluctuation of the complex, so that the applied load has both tensional and transverse components. Adapted from [76].

To define the origins of the force responsiveness of the αβTCR, it is necessary to probe T cells and T‐cell associated proteins with single‐molecule techniques capable of generating and resolving pN forces including, but not limited to, OT‐based single molecule (SM) [31, 35], single molecule‐single cell (SMSC) [31, 63], single cell activation requirement (SCAR) [32, 63], as well as molecular dynamics (MD) simulation [61, 76]. Other technologies, not elaborated herein, have been used for a similar purpose, such as BFP [30, 51], atomic force microscopy [77], traction force microscopy [78, 79], and bead‐based thermal force generation [80]. Figure 4 illustrates the biophysical techniques commonly used in our laboratories. To interrogate the receptor proteins directly, the SM assay [31, 35] (Figure 4A) immobilizes pMHC via a biotin‐streptavidin linkage on a PEG‐passivated glass slide attached to a piezoelectric‐driven stage. This provides a movable proxy for the APC, allowing sub‐nanometer precision in the motion of the stage relative to the TCRαβ. The TCRαβ itself is held in an OT by attachment to a polystyrene bead via serial linkage from the bead to the TCRαβ. The serial linkage to the TCRαβ begins with an anti‐digoxygenin monoclonal antibody (mAb) bound to digoxygenin. The digoxygenin is covalently linked to an extended double‐stranded DNA oligonucleotide, which in turn is covalently attached to the anti‐leucine zipper (LZ) 2H11 antibody. This antibody binds to the C‐terminal LZ pair tag ensuring proper pairing of the TCRαβ ectodomain heterodimer [81, 82]. By moving the pMHC‐functionalized piezostage relative to a trapped TCRαβ‐coupled bead, one can discern the binding of TCRαβ to pMHC by displacement of the bead from the center of the optical trap (ΔX in Figure 4A). The stage can then be moved a precise distance, further displacing the bead from the trap. This bead displacement correlates with the force applied to the system in series. The bead in the trap functions like a spring, with its stiffness calibrated for each assay [83, 84]. By varying the force on the TCRαβ‐pMHC bond, one is able to collect data revealing the catch bond described above (Figure 3B); The bond lifetime increases with force up to a critical point, or optimal force, and it decreases thereafter. Maximum ligand discrimination and transitioning implicated in signaling occurs within the optimal force regime [31, 35, 60]. By replacing single components of the system such as the peptide, the pMHC, or the TCRαβ, one is able to discern the specificity of the interaction in terms of catch bond strength. Moreover, by adding exogenous factors like mAbs specific for regions within the TCRαβ, one can discern allosteric contributions of those regions. Since the remaining components of the force pathway are constant, granular dissection of the receptors and ligands is possible. This is true also for the conformational changes (Figure 3C,D) observed within the TCRαβ and the pTα‐β, which were shown to vary with ligand identity or site‐specific antibodies, such as the binding of the fragment antigen‐binding (Fab) of the H57 mAb to the Cβ FG loop [31, 35]. While critical forces were similar, the transition frequency was greater for the N15 preTCR compared to the N15 TCRαβ when bound to the same pMHC ligand in identical experimental conditions, suggesting that the compliance was greater and potentially the signal initiation threshold is lower for the preTCR [35].

The second assay which has generated critical data is the SMSC (Figure 4B). It allows interrogation of the biophysical parameters of the αβTCR in its native state on the cell surface, including all CD3 components as well as other surface proteins, and, in this respect, is akin to the BFP assay [30, 31]. In contrast to the SM assay, in the SMSC assay the pMHC is linked to the OT‐bound bead via a C‐terminal biotin, anti‐biotin mAb, DNA oligonucleotide, digoxygenin and anti‐digoxygenin mAb, while the T cells bearing the αβTCR of interest are arrayed in a microchamber on the piezostage. As such, the stage is moved until a bead is displaced, after which the stage is further moved until a trap displacement is attained with the desired amount of force. Catch bond formation and conformational transitions were confirmed by the SMSC assay, highlighting the relevance of the SM measurements in exploring mechanotransduction [31]. This experimental design has allowed us to ask questions not only of the mechanisms within the ectodomains, but also to probe relationships among components of the full holocomplex [63].

The SCAR assay [Figure 4C; [32]] utilizes a bead functionalized with quantitatively controlled pMHC interfacial density that is approximated to a T cell. The parameters for activation of the T cell can be determined using SCAR by applying a controlled force during binding. This experiment uses an instrument with OTs capable of trapping simultaneous with fluorescence detection [85]. In this case the pMHC is directly coupled to bead‐bound streptavidin without a DNA oligonucleotide linker, thereby reducing compliance in the system and allowing general control over directionality of applied force of the multitude of bead‐bound pMHC molecules. T‐cell activation is detected by increases in intracellular calcium via fluorescence monitoring over time. This allows for the examination of parameters including pMHC specificity, force optima for activation, activation kinetics and pMHC copy number thresholds [32, 60, 63]. This assay has been instrumental in defining the differences between digital and analog αβTCRs with activation of αβT cells detected to a pMHC interfacial copy number of two. But the requisite number in vivo is probably just one in reality, given the absence of any adhesion molecule or co‐receptors on the polystyrene bead array.

The most recent development is dual‐trap SM assay (Figure 4D) which replaces the slide‐bound component of the SM assay with a second, movable OT [60]. This experimental geometry features increased spatial resolution relative to the standard SM, and increased control of forces applied to the TCRαβ‐pMHC bond. As discussed below, this innovation enabled the capture of resonant states of the TCRαβ‐pMHC interaction, allowing for a folding‐unfolding transition that achieved bond lifetimes on the order of minutes [60].

In addition to SM experiments, all‐atom MD simulation can be used to reveal dynamic mechanisms of the TCRαβ‐pMHC interaction. Here pN‐level load is applied via harmonic constraints on C_α_ atoms of residues at the ends of the complex [pink dots in Figure 4E; [61, 76, 86]]. Force is measured via deviations of those atoms from centers of restraining potentials, analogous to an OT experiment [87]. Although the μs time scale of the simulation is insufficient to observe the full bond lifetime that extends over several seconds, since force propagates at the speed of sound through a protein [88], it is enough time to observe how the conformational dynamics of the complex is altered by load in a ligand‐dependent manner. There is also the potential to deconstruct the system, removing or altering residues, loops or domains to ask questions about the role, for example, of the subdomains of TCRαβ for catch bond formation [61, 76, 86].

### 2.4 Sources of Force, Internal and External

T cells scan their environment using the force from the constantly rearranging cytoskeletal matrix (Figure 3A and Videos S1 and S2). The force provides the anchor and pull necessary to activate the mechanical properties of the αβTCR. On a macroscopic level, the αβT cell experiences starts and stops, leading to the generation of significant forces [79, 89]. The αβTCR itself is clustered in microvilli that are rich in cytoskeletal elements at the leading edge of scanning αβT cells that dynamically polymerize while myosin motors exert forces on them [90]. Indeed, proper αβTCR activation requires cytoskeletal connection [26, 32], although the clustering of αβTCRs within the microvilli after the initial pMHC recognition may not require cytoskeletal connection [34]. Connection to the cytoskeleton was evident in the positional relaxation of the bead in the optical trap upon αβT‐cell activation in SCAR assays. With the application of tangential force onto a T cell using cognate pMHC, the trapped bead regressed towards the center of the trap with discrete ~8 nm steps. Myosin inhibitor blebbistatin and actin depolymerizer cytochalasin D disrupted this discrete relaxation significantly, but microtubule depolymerizer nocodazole did not [32]. While administration of all three compounds in this study abrogated T‐cell signaling due to the general role of the cytoskeleton in activation, the above results illuminated a specific mechanism in the connection of the αβTCR to actomyosin.

Following the initial activation of the T cell, the formation of the immunological synapse occurs. By monitoring αβTCR complexes on the same cell that were not directly bound to pMHCs, but rather were “bystanders” to activation, it was found that an activation event set up a transit network to move unligated αβTCRs towards the triggered αβTCR. This transit of micrometer distances in response to activation may represent the first step towards the initiation of the immunologic synapse and the internalization of αβTCRs, which subsequently down‐regulates surface levels after activation, highlighting the complexities of αβTCR‐cytoskeletal engagement [32]. The immunologic synapse forms in the uropod of the T cell, requiring active transport of the αβTCRs to the trailing edge of the cell [57]. This transport is essential for maintaining prolonged activation of the T cell as it stays attached to the APC. These interactions last for minutes and even hours with apparently differential consequences depending on the duration of interaction for both CD8 and CD4 T cells [91, 92, 93, 94, 95, 96]. The role of forces on the individual receptor‐ligand pairs at these longer timescales, including αβTCR‐pMHC, as well as other cell‐adhesion molecules may vary significantly from the initiating events that we have described thus far.

More quantitatively, forces generated at the T‐cell‐APC interface can be directly measured. First and foremost are the measurements of forces exerted on individual αβTCR‐pMHC pairs using intact T cells. SMSC experiments generally agree with SM measurements, putting the optimal catch bond forces from 10 to 20 pN for maximal bond lifetimes [31, 32, 35]. In rare cases forces exceed 20 pN on a αβTCR‐specific basis in either assay [60]. These measurements are bolstered by the SCAR measurements placing the force maxima in the same magnitudes, with optimal force exerted by presumed single bonds at limiting pMHC numbers in the range of 10–15 pN. Those measurements are in broad agreement with others in disparate systems [30, 59], suggesting there is ample force at the T‐cell interface. A caveat is that interfacial forces are measured for multiple interactions and at different temporal points relative to initial activation. Cantilever‐actuated atomic force microscopy has been used effectively in other systems, but the nN‐range forces are several orders of magnitude greater than the optimal level for T‐cell activation [77]. Such measurements may be more appropriate to uncovering junctional contacts between αβTCR and APC following activation [78, 97, 98]. Meanwhile, systems relying on synthetic pillar deflection to measure traction forces show 100‐pN forces exerted on CD28 and CD3, though the pMHC levels in these systems are high, resulting in multiple bonds per measurement [78, 99]. A study directly comparing forces generated from fibroblasts to activated Th1 cells demonstrated that traction stresses were an order of magnitude less than those of fibroblasts, in the hundreds of Pascals. While forces from fibroblasts were mainly contractile, for Th1 cells the distribution of forces spanned all angles including contractile and pushing directions [79]. Furthermore, the force exerted on a single pMHC in a variety of measurement geometries via fluorescence‐based DNA hairpin sensors is also in the range of 10–20 pN [59]. DNA hairpin technology has recently been used to investigate the force pathway on the APC side of the equation ([100] and see below).

Exertion of force and displacement suggests energy consuming features of pMHC recognition and T‐cell activation. The reversible transitioning (Figure 3C,D) involves free energy consumption that approximately equals the hydrolysis of 2 ATP molecules [101] which is about 40 times thermal energy (referred to as k_B_T, thermal energy is provided by random fluctuation of the surrounding solvent molecules and it is equal to 4.3 pN*nm at 37°C). Need for such a large amount of free energy can be understood based on the quaternary structure where the energetically driven transitioning on the ectodomain can agitate the plasma membrane, rearrange the TM domains, then “drum up” ITAM‐containing cytoplasmic tails to allow phosphorylation by Lck. These series of changes occur in a coordinated way over lengths spanning well over several nanometers within the strongly dissipative environment imposed by the lipid bilayer as well as crowding conditions of the cytoplasm. It is unlikely that mere binding of a cognate pMHC by itself can trigger such large‐scale changes. Requirement of free energy consumption may also serve to guard against autoreactivity where transient interactions with numerous self‐pMHCs are unlikely to drive sustained reversible transitioning.

The load connecting pathway is coupled to and terminates at the underlying cytoskeletal structure. While a relatively short segment of an isolated actin filament can readily bend ~10 nm with thermal energy, actin binding proteins including crosslinking agents help to stabilize these structures. The strength of this structure is complex and depends on the number of filaments, their relative geometry and degree of crosslinking. Rupture forces for individual actin binding proteins such as filamin and α‐actinin are on the order of 40–80 pN [102] suggesting even a modestly developed cytoskeletal network will be able to sustain load to anchor the αβTCR.

### 2.5 Forces Generated on APCs and Association of pMHC With Cytoskeletal Matrix

Interactions of MHC molecules and actin were first suggested by studies showing that exfoliation of exosomes, possibly microvilli, results in detergent‐stable complexes of actin and murine MHC (H‐2) [103]. While attempts were made to understand the membrane and cytoskeletal anchoring of MHC [104, 105, 106, 107, 108, 109, 110], the molecular mechanism of MHC engagement with the cytoskeleton is still unclear. In dendritic cells, the MHCII molecules are trafficked to the cell surface via endocytic vesicles in a maturation dependent manner [111], and are trafficked to the site of interacting T cells via tubulation [112]. The patch‐like distribution of MHC may be maintained during this process through regulation of relative delivery and internalization rates as well as diffusion hindered by the actin cytoskeleton [113]. Other studies have demonstrated an interaction between myosin II and MHCII via the endoplasmic reticulum‐resident protein invariant chain (Ii; CD74), connecting MHC clustering to the actomyosin cytoskeleton and identifying the source of the physical interaction for some systems [114]. On the other hand, studies on the mechanical activation of T cells via MHC generally rely on MHC anchored to functionalized rigid surfaces [59], within membranes on flat surfaces [98, 115, 116], to beads in optical traps [27] or to beads affixed to cellular surfaces [30]. Recent work has measured αβTCR‐pMHC forces at cellular interfaces using membrane‐embedded DNA origami nanosensors coupled to pMHC on B‐cell surfaces to be > 8 pN [100]. While these experiments are tantalizing and represent a lower bound to the forces generated from APCs, the membrane attachment and local native compliance of the authentic MHC TMs and cytoplasmic regions will be necessary to put the final piece of the story together. It will be advantageous to characterize the response of MHC molecules under force, including the typical force resistance profiles and how these may vary with cell types, maturation and activation states, and any differences between classes of MHCI and MHCII molecules. This analysis should utilize native molecules embedded within membranes, although such experiments may be challenging at present. A more precise structural definition of MHC intracellular associations will also be needed to fully understand the contribution of APCs to the activation of T cells.

### 2.6 Molecular Mechanisms of Mechanotransduction and Signaling

Forces converge in series from the T cell to the APC, connecting the cytoskeletal matrix of one to that of the other. For the ectodomains, the system geometry must align with the configuration depicted in Figure 2D, where loads have axial and shear components. In CD8 T cells the force is funneled through the TCR G‐strands of the respective C domains, converging on the disulfide linkage proximal to the membrane and preceding CP regions. The force path on the MHCI side converges on the CP region and TM of the heavy chain since β2m possesses no membrane anchor. The forces are approximated by the harmonic restraints applied at terminal strands in MD simulation studies [61, 76, 86], where the added strands partially incorporate the presence of CPs (Figure 4E). Similarly, the force paths generated in the SM and dual trap SM mimic the ectodomain anchoring, (Figure 4A,D), whereas SCAR and SMSC substitute the full αβTCR holocomplex anchored by their native CPs and TMs, adding more physiologic relevance to the system while preserving the pMHC anchor (Figure 4B,C). The concordance in MD and biophysical wet lab design has facilitated significant sharing of ideas and crucial reality checks [37, 58].

**FIGURE 5:**
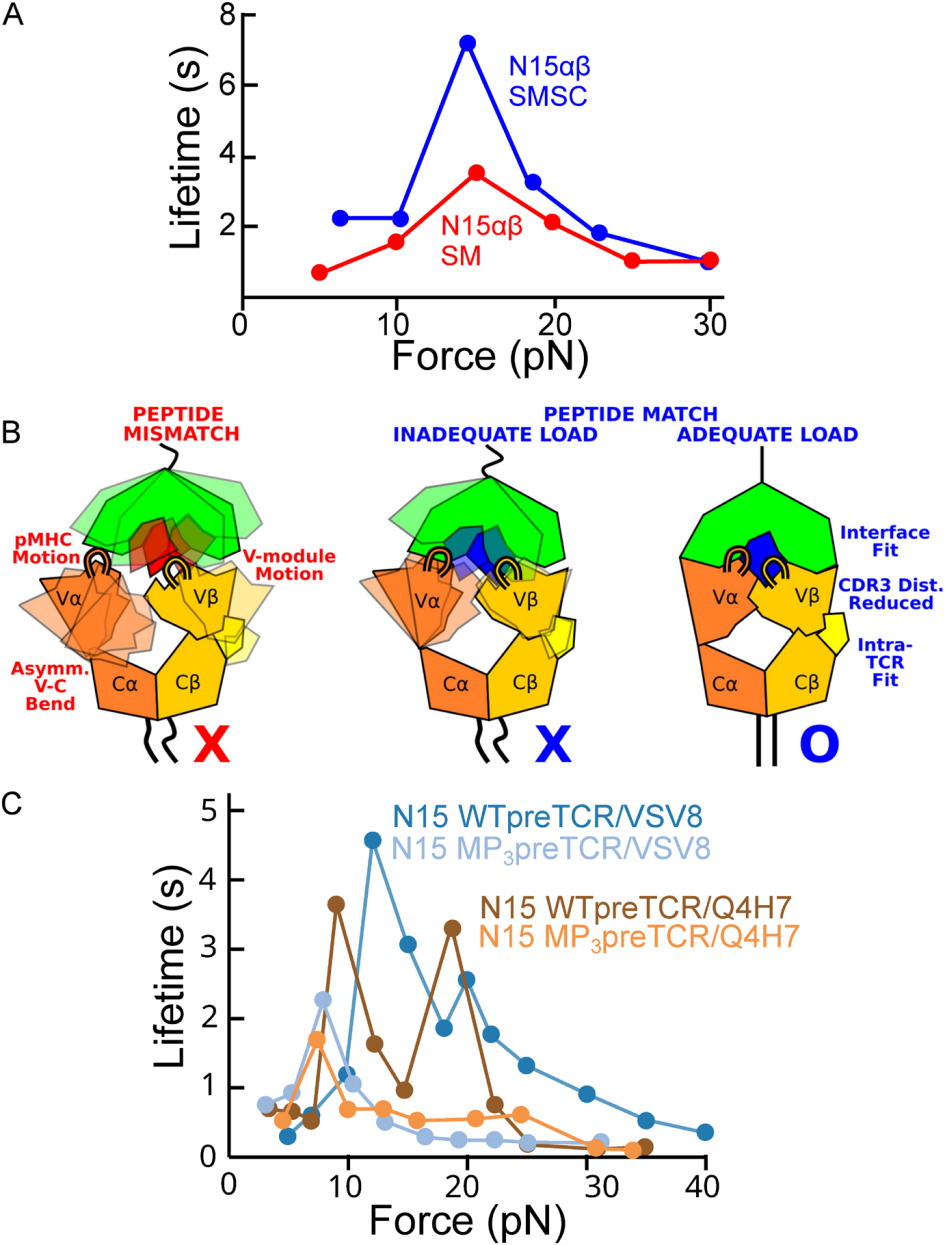
Measuring the relationship between force and the stability of the TCRαβ‐pMHC and preTCR‐pMHC interactions. (A) Force‐bond lifetime relationship for isolated N15 TCRαβ ectodomains (red trace) or αβTCR holoreceptor (blue trace) in binding VSV8/K^b^ using SM or SMSC, respectively. SMSC data is derived from experiments using an α3 mutant K^b^ molecule which does not bind CD8 to directly compare only TCRαβ‐pMHC interaction between experiments. Plots have been simplified for clarity. Panel adapted from [31]. (B) Atomistic basis for load sensitivity of the TCR. Due to the uneven compliance of the interfaces between the 4 subdomains, Vα moves more than Vβ does relative to the C‐module. Without an adequate load or the matching peptide, the asymmetric motion of the TCR chassis destabilizes the interface with pMHC (left). When a proper amount of load is applied while a cognate pMHC is bound, the subdomain motion can be suppressed and interfaces can be stabilized (right), which is the basis for ligand‐dependent catch bond formation. Panel adapted from [61]. (C) Force‐bond lifetime relationship for wild‐type (WT, dark shaded traces) or Vβ patch mutant (MP_3_, light shaded traces) N15 preTCR in binding VSV8/K^b^ (blue traces) or Q4H7/K^b^ (brown traces) using SM. Plots have been simplified for clarity. Panel adapted from [35].

Foremost is the observation of the catch bond itself. First measured for αβTCRs via BFP [30], the catch bond was confirmed to be a property primarily of the ectodomain using SM as shown for the N15αβ‐VSV8/K^b^ system that forms a catch bond with a peak lifetime of approximately 4 s at 15 pN [Figure 5A; [31]. It was also noted that when the same clonotypic receptor was interrogated with SMSC, the full receptor displayed a ~twofold longer lifetime [Figure 5A; [31]. The ectodomain origin of the catch bond allowed examination via all‐atom MD (Figures 4E and 5B). The first salient observation was that applying force on the interaction pair effectively dampened inter‐subunit motions, preserving interatomic contacts at the TCRαβ‐pMHC interface [Figure 5B; [76]]. This motion‐based disruption of the binding interface is insufficiently dampened without force and can be modulated by the peptide itself, providing the rationale for synergistic gating of both peptide and force for T‐cell specificity [61, 76, 86]. Intriguingly, the interface between the Vβ and Cβ domains was found to be robustly coupled in simulations due to the presence of the Cβ FG loop, an approximately 12 amino acid residue insert unique to αβTCRs in jawed vertebrates among Ig‐like proteins [8, 36, 49, 76]. Moreover, deletion of the Cβ FG loop (ΔFG) in MD led to substantial weakening of the interface under force. This resulted from altered V‐C bend angles and attendant changes in conformational motion of the Vα‐Vβ interface, disrupting the TCR‐pMHC interface as force is applied [61, 76]. This finding agrees with the enhanced dissociation of the ΔFG variant of the N15αβ TCR in SM and SMSC experiments when force is applied [31]. On the other hand, stabilizing this loop by the binding of the Cβ FG loop‐specific H57 Fab increases SM‐measured bond lifetimes by 10‐fold [31]. This observation agrees with the deleterious effects to T‐cell signaling and developmental progression when the FG loop is excised from the N15αβ TCR in transgenic mouse experiments [117]. The domain motions also appear dampened, albeit to a lesser extent, with the addition of the anti‐Cα H28 Fab [31], implying that the domain crosstalk extends to all other regions of the protein (Figure 5B).

It remains unclear if the crosstalk is an effect engendered directly through the Vα‐Cα interface or indirectly via stabilized Cα‐Cβ which could then influence the Vβ‐Cβ interface and ultimately affect TCR‐pMHC binding. The V‐C interface of the αβTCR is certainly unique in that neither antibodies (or B‐cell receptors; BCRs) nor γδTCRs, as will be discussed later, possess similar coupling between V and C domains, with the homologous interface to Vβ‐Cβ in antibodies having approximately half the buried surface area [31, 36]. The strong influence β has on the catch bond is evident in the function of the preTCR, in which the pTα ectodomain lacks a canonical variable domain (Figure 1C). Like the TCR, the preTCR forms a seconds‐long catch bond with pMHC (Figure 5C), and this is dependent on the Cβ FG loop, as H57 Fab stabilizes the catch bond by 10‐fold while the ΔFG mutant binds ligand with a slip bond fashion and loses the ability to discriminate ligands [35, 51]. What differentiates the preTCR from the αβTCR is the relaxed ligand specificity of the former. In the two preTCRs tested against the cognate ligand of the mature αβTCR, there was a significant catch bond when interrogated with the pMHC, VSV8/K^b^, and also cross‐reactivity against two unrelated peptides bound to the same MHC, OVA(seq:SIINFEKL) or Q4H7(seq:SIIQFEHL)/K^b^, but not SEV9(seq:FAPGNYPAL)/K^b^ [35].

The other difference noted was the complexity of the bond‐lifetime curve for most ligands. The N15 preTCR‐SEV9/K^b^ binding is dominantly a slip bond, where the lifetime decreases monotonically with force, but the N15 preTCR‐VSV8/K^b^ binding showed a strong catch bond at ~12 pN and a second, shorter catch bond at 20 pN (Figure 5C). For Q4H7/K^b^, two catch bonds of nearly equal lifetimes at 10 and 20 pN were evident [Figure 5C; [35]]. Ongoing investigations also reveal complexities in catch bond profiles of mature αβTCRs, where different clonotypes exhibit varying optimal forces [60] and shifting of force‐lifetime profiles through mutations in the TCRα TM [63]. For the preTCR, mutagenesis of Vβ‐patch residues that interact with MHC residues (Figure 3A,B) in the N15 preTCR‐VSV8 or N15 preTCR‐Q4H7/K^b^ complexes abrogated the high‐force (20 pN) catch bond (Figure 5C). This also led to a reduction in the bond‐lifetime by approximately half and decreased the force maximum of the remaining catch bond by 2–4 pN (Figure 5C) [35]. The precise mechanism behind this complexity remains unknown. We posit that the Vβ subunit in the preTCR as compared to its position within the TCRαβ when binding pMHC may permit the second catch bond. This may be due to force‐induced changes in Vβ or to selection of alternate binding orientations. Future detailing of load pathways shall test the veracity of this idea.

The second major observation regarding αβTCR mechanotransduction was of a structural transition within the ectodomain of TCRαβ with force [Figure 3C,D; [31]]. The transition, like the catch bond, is largely β‐dependent, as it is abolished upon stabilization of the Cβ FG loop with the H57 Fab, accelerated in ΔFG TCR, and it is also present in the preTCR which lacks the α chain [31, 35]. In contrast, the transition is not observed in the γδTCR interacting with its ligand unless the Cαβ domains are swapped for the Cγδ domains, and are then actuated in a ligand dependent manner [36]. The transition is on the order of 10 nm and importantly, is reversible [35, 60]. It is plausible that certain domains of the αβTCR unfold, analogous to the mechanical unfolding of titin, which also has an Ig fold [118]. Instead of providing elastic energy to assist in muscular contraction, the αβTCR may utilize the energy harvested from mechanical force to initiate structural rearrangements to allow phosphorylation of ITAMs. The precise nature of the structural transitions is yet to be elucidated. While evidence indicates that the transition is largely resident within the ectodomains of TCRαβ, the cryoEM structures point to additional components dependent upon the CP, TM, and the associations between CD3 and TCRαβ ectodomains, as well as with the lipid bilayer [17]. That study reveals the squat unligated holoreceptor to have compacted CPs and TMs, with the latter far from straight helical configurations, then transitioning to uncompacted and extended structures, respectively, following pMHC ligation and an associated ectodomain extension. How the cryoEM observed changes per se relate to the transitioning and measurably larger extensions characterized by OT requires further study.

We have recently observed a phenomenon termed ‘volleying’, which is characterized by rapid (up to 40 Hz) transitioning repeatedly for minutes at a time [60]. Volleying appeared as an extension of the previously observed transitioning [31, 35, 60], with the continued transitioning resulting from a more precise control of forces in the dual‐trap assay allowing the TCRαβ itself to remain near the equilibrium force where it is equally likely to be in the folded (compact) or unfolded (extended) state (Figure 3C). The significance of volleying is tied to the amount of energy that can be redirected from the externally generated force to the surrounding molecules, including CD3 and the lipid bilayer. The repeated transitioning associated with a single bond could generate sufficient energy to dissociate CD3 domains noted below, dislodge the ITAMs from the inner leaflet of the plasma membrane, and initiate a phosphorylation cascade within seconds of binding (Figure 3C). Given that catch bonds can last seconds to tens of seconds as experimentally determined, with volleying lasting minutes or more, amplifying a single bond to a full signaling cascade is possible. Furthermore, while it is natural to think that bond lifetime drives signaling, our perspective is that the force on the bond energizes states that permit access to downstream signaling pathways. The probability of accessing these pathways depends exponentially on the energy available relative to the energy that is required to enter that pathway.

### 2.7 Dynamic Role of CPs and TMs in αβTCR Signaling

**FIGURE 6:**
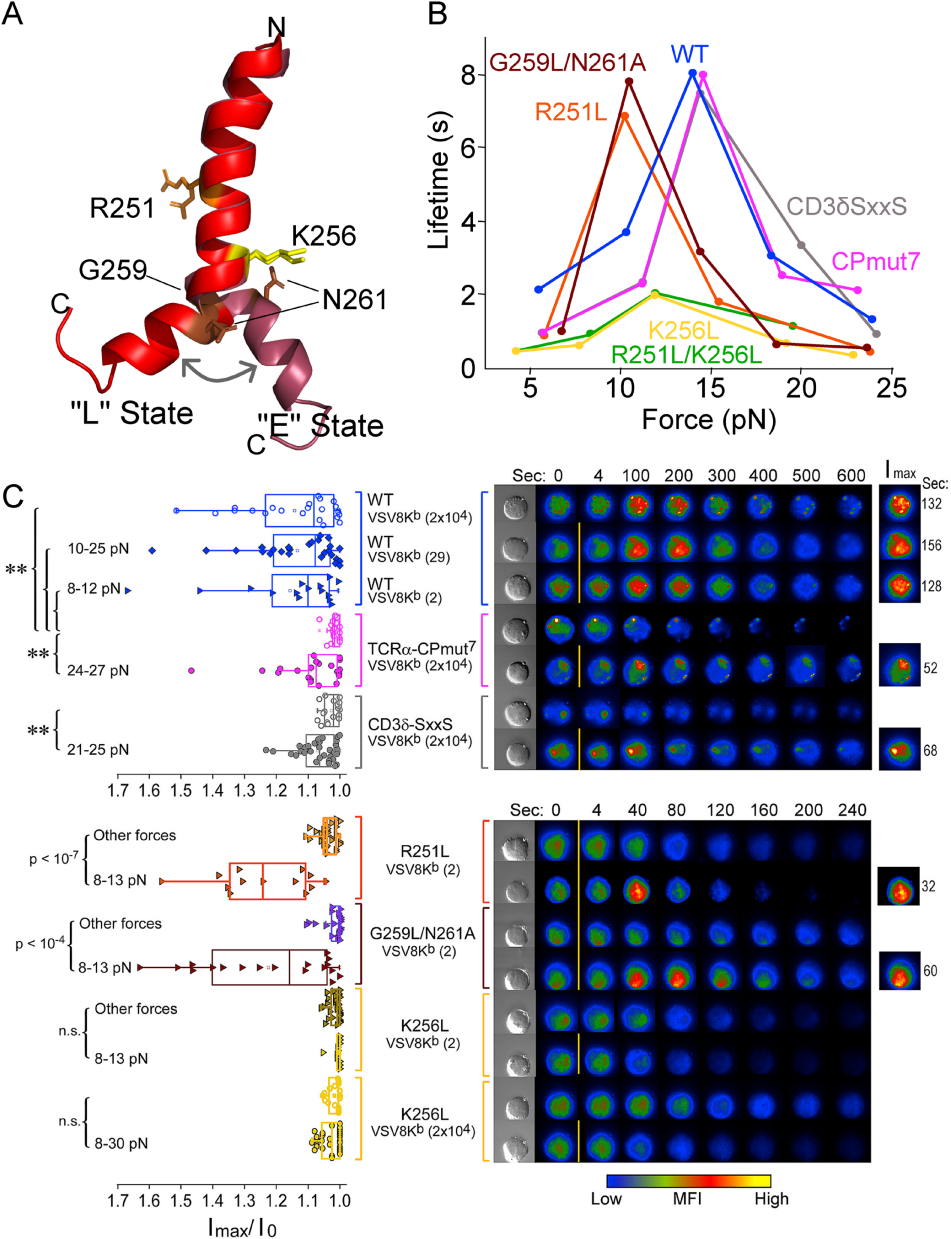
TCRα transmembrane and connecting peptide structure directly impacts parameters of mechanosensing. (A) NMR structures of two conformers of TCRα TM region showing “L” state (red) and “E” state (raspberry). Residues important for TCR function are shown in sticks. (B) Bond‐lifetime vs. force curves of SMSC measurement of TCRα TM and TCRα and CD3δ CP mutants as compared to the WT N15αβTCR. Plots have been simplified from the original for clarity. (C) SCAR assay of mutants assayed in SMSC in panel B. Left: Plots of relative maximum fluorescence intensity with triggering (I_max_/I_0_) with each symbol representing a single cell measured. Right: Representative triggering trace and the fluorescence image at maximum fluorescence (I_max_) and the time for which it occurred. Each mutant is shown at high pMHC copy number (2 × 10^4^ interfacial pMHC) and at lowest pMHC copy number in which a response was seen and at optimal force needed to trigger. All are compared in relation to the WT N15αβ TCR. Figure adapted from [63].

As discussed above, the CP and TM regions of the TCRαβ and the associated CD3 components couple the extracellular regions to the cytoplasmic tails for signaling and, presumably, cytoskeletal attachment. The TMs are not simply helical membrane anchors for the αβTCR. Figure 6A illustrates structures of TCRα TM as characterized with NMR and electron paramagnetic resonance spectroscopy (EPR) [63]. This membrane spanning region features a conserved sequence that drives dynamic conformational heterogeneity that has functional implications. In particular, the conserved Lys (αK256), previously recognized mainly for charge‐mediated TM bundle organization [68], regulates TM immersion depth or angle of the TM, and acts as a master controller of CD3 dissociation upon αβTCR activation [63]. The K256L mutation in T cells severely diminishes αβTCR catch bond formation (Figure 6B, yellow curve), leads to CD3 dissociation from the αβTCR, and abrogates T‐cell triggering as measured by SCAR [Figure 6C; [63]]. Yet, this hypo‐responsiveness of the K256L mutant in the SCAR experiment appears to be a late, long‐term effect of the mutation since cells harboring this mutation bear the hallmarks of a strongly activated T cell in transcriptome analysis. Furthermore, the dissociation of CD3 from the αβTCR is implicated as an intermediate in T‐cell stimulation processes, underscoring the impact of this mutation on immune response [63].

The abrogation of the catch bond with the K256L mutation (Figure 6B) is intriguing as it is consistent with a change in the TM positioning in the plasma membrane, TCRαβ association with CD3 signaling dimers, or both. The other TM mutations tested with SMSC, R251L, mutating the second conserved intramembrane charge, implicated in CD3ζζ association, and the G259L/N261A double mutant which forces the TCRαTM to populate only the extended state, resulted in similar catch bond lifetimes but a lower force maximum (Figure 6B, dark and light red curves). These mutations in the TM domain may result in different orientations of the TCRαβ ectodomain, thereby affecting the loading direction when bound to pMHC and catch bond behavior, similarly as shown for the Cβ FG loop deletion mutant in all‐atom MD simulations [61, 76]. While the mechanism of these effects warrants further investigation, this functional phenotype suggests that the intersubunit associations are weakened, leading to more compliance in the system and reducing the amount of force necessary and tolerated for a catch bond. In some of these mutant cell lines tested with the SCAR assay, the T cell is triggered readily, yet the triggering does not result in as much sustained signaling as the wild‐type cell line (Figure 6C), possibly because the cells are already in an activated state due to disassociation of CD3ζζ as shown for the R251L T‐cell line [63]. Mutation of CP regions corresponding to the site of association between CD3δ and TCRα [15, 63] did not strongly affect the catch bond curve measured by SMSC (Figure 6B). However, it did show a notable decrease in signaling potential in SCAR assays. This resulted in a loss of digital sensitivity of the N15 αβTCR, which only triggers at higher forces and very high pMHC copy number, and even then, it activated weakly (Figure 6C). These results inform that the pathways of load propagation and signal transduction are intricately related while they cannot be considered identical. This is evident in the case of αK256 as a master controller of activation. αK256 influences both the potential for catch bond formation, possibly by affecting membrane positioning or TM associations, and the triggering process. This can be seen by the K256L mutation that leads to dissolution of the αβTCR complex (i.e., loss of CD3), leading to an activated T cell that cannot be further triggered due to loss of these signaling subunits. Whether the K256L mutated heterodimer requires CD3 components during assembly with disassociation occurring after the αβTCR reaches the surface or whether TCRαβ can reach the surface independently of CD3 is unknown at present.

There is still much to be understood about the complex roles of the CPs and TMs in T‐cell signaling. Other laboratories have supported the mechanism of CD3 disassociation in αβTCR triggering [119, 120]. It will be valuable to accurately track in future studies the sequence of events leading to disassociation under native pMHC ligation and whether other TM regions may play a key role for this process. The weaker effects of the αR251L and G259L/N261A mutations suggest disassociation of CD3ζζ may be a less stringent or early intermediate in the activation mechanism, implying that understanding the web of complex interactions within the αβTCR holocomplex will require more careful analysis.

## 3 Mechanosensing and Biological Roles of T Cells

### 3.1 αβTCRs Within a Single Repertoire Are Functionally Divergent and Their Affinity‐Function Relationship Is Not Monotonic

**FIGURE 7:**
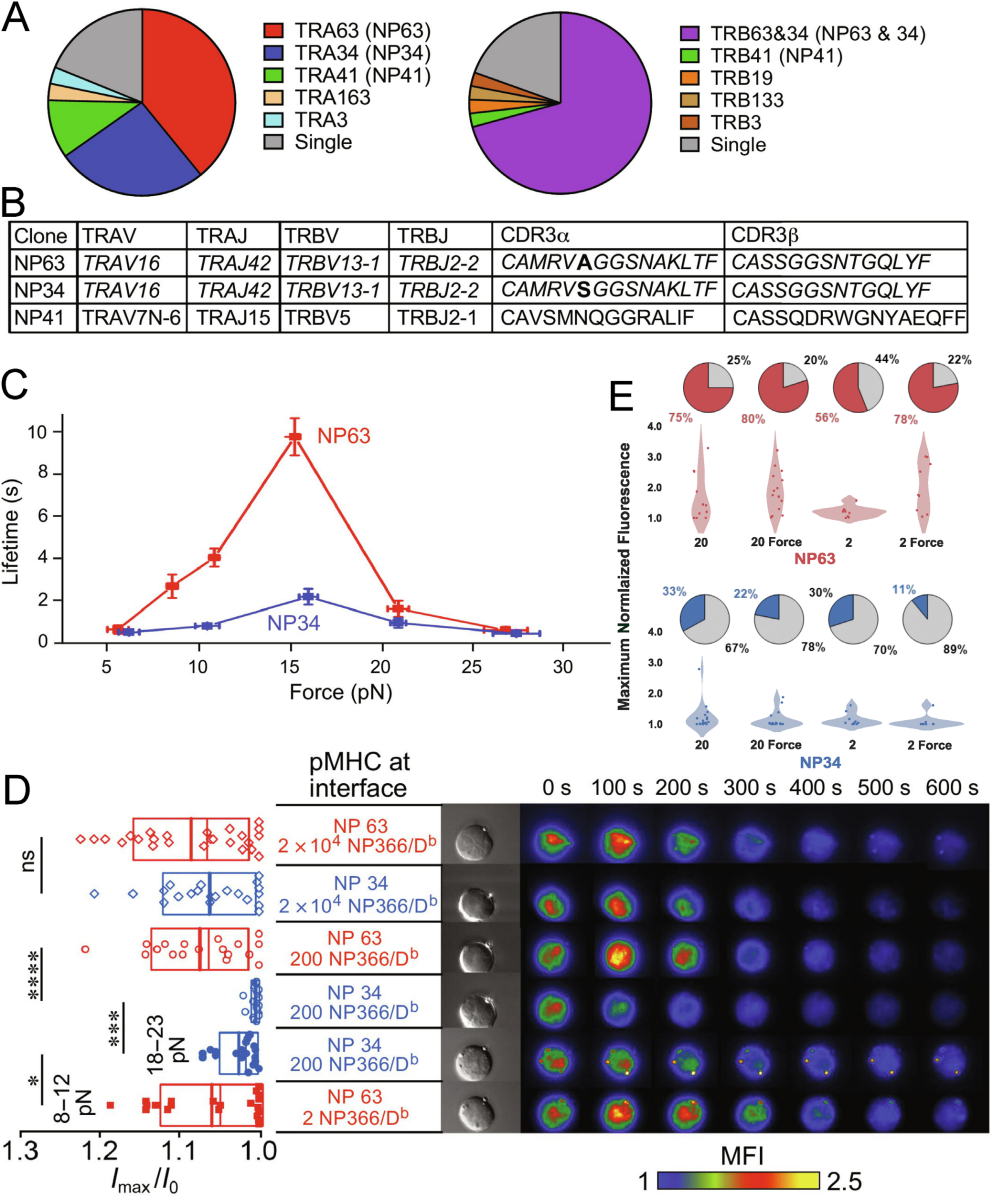
Differential sensitivity of highly related TCRs specific for high‐copy ligand NP_366‐374_/D^b^ display in mice challenged with IAV infection. (A) Proportions of V gene usage by NP_366‐374_/D^b^ specific T cells in recall responses to IAV. The top two TCRs (NP63 and NP34) account for the majority of NP_366‐374_/D^b^‐specific clonotypes. (B) Gene usage for top three most common clonotypic TCRs as well as translation of CDR3 regions for TCRα and TCRβ. Note the near identity of NP34 and NP63. (C) SMSC Force‐lifetime plots of NP34 and NP63. (D) SCAR profiles of NP34 and NP63. Left: Plots of relative maximum fluorescence intensity with triggering of individual cells given as symbols (I_max_/I_0_). Right: Representative triggered trace for each. Each TCR is shown at high pMHC copy number (2 × 10^4^ interfacial pMHC) and at minimum number in which a response was seen and at optimal force needed to trigger. (E) Violin plots depicting the maximum normalized fluorescence of each tested cell for NP63 and NP34 after pMHC stimulation. Interfacial numbers of pMHC are shown below the plots. Pie charts show triggering percentage. Solid color represents percentage triggered, gray color represents non‐triggered. A recovery of triggering percentage and maximum normalized fluorescence is observed in NP63 for two interfacial pMHC when force is applied to the TCR‐pMHC bond, confirming its digital capability. However, NP34 shows no recovery with force application, indicating it is analog. Figure adapted from [60].

It is increasingly clear that clonotypes dictate function and fitness of αβTCRs and that their fitness depends on factors beyond affinity. When selecting an αβTCR clonotype for transfer into patients aimed at treating cancer or other diseases, it is crucial to carefully evaluate individual αβTCRs with their ligands to ensure maximal efficacy against a pMHC target. Within a repertoire of T cells, all specific for the identical pMHC of an influenza A virus (IAV) Puerto Rico 8 (PR8) strain, there appears to be a differential of efficacy even among highly similar αβTCRs isolated from the memory compartment after secondary challenge with IAV [60]. Approximately two‐thirds of T cells in the expanded pool specific for NP_366‐374_/D^b^ were comprised of two clonotypes, each utilizing an identical β subunit with only a single residue difference within CDR3α (Ala to Ser) (Figure 7A,B). The expectation was that these two αβTCRs, termed NP34 and NP63, being identical except for a single CDR3α residue, would be functionally identical. Yet SMSC showed that there was a five‐fold (10 versus 2 s) differential in maximal catch bond lifetime at 15 pN optimal force of clonotype NP63 versus NP34 (Figure 7C). Despite equivalent IL‐2 response to peptide titration in the standard cellular assay, NP63 was also found to be superior when assayed by SCAR at limiting peptide concentration (Figure 7D,E). NP63 also showed rescue of activation at 2 interfacial pMHC in the presence of force through intensity of calcium flux and percentage of cells triggered (Figure 7E). This correlated well with other cellular assays including CD69 upregulation, ERK phosphorylation, and in vitro cell killing [60]. Thus, like the N15 αβTCR recognition of VSV8/K^b^, NP_366‐374_/D^b^ recognition by NP63 but not NP34 is digital, requiring as few as two pMHC with force in the SCAR assay [32, 60] despite differing by only a single residue.

**FIGURE 8:**
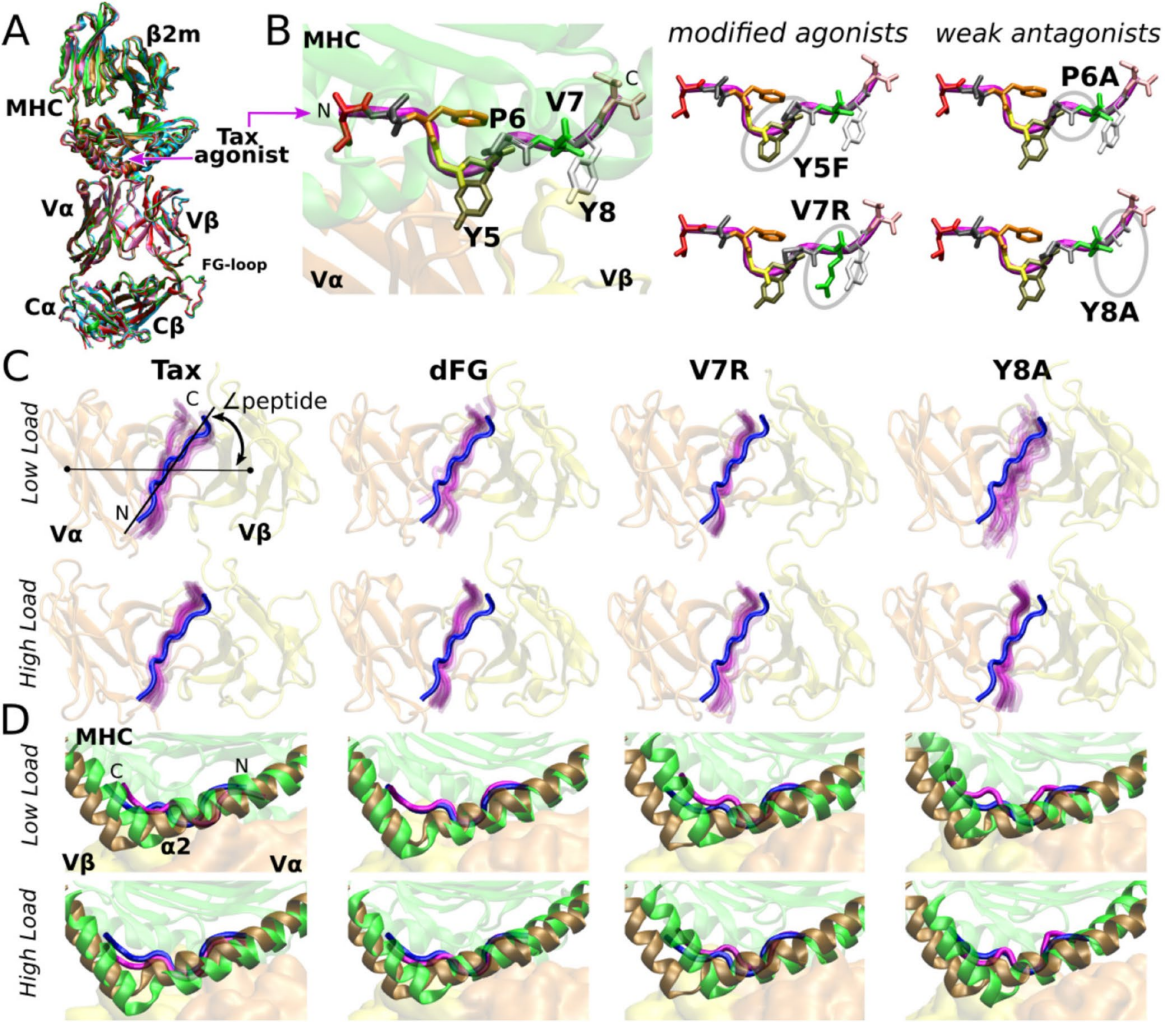
MD simulations show point mutations of the Tax agonist peptide bound to A6 TCR alter the TCR‐pMHC interface in a load‐dependent manner. (A) Overlay of five A6 TCR‐pMHC crystal structures with different bound peptides: Wild‐type Tax agonist [brown, PDB 1AO7, [12]], Y5F [red, PDB 3QFJ, [122]], V7R [pink, PDB 1QSE, [123]], P6A [cyan, PDB 1QRN, [123]], and Y8A [green, PDB 1QSF, [123]]. Note all 5 structures align very closely. (B) Tax peptide residues from crystal structures are shown in licorice representation. Point mutations included in MD study are labeled. The peptide conformations are essentially identical even though point mutations lead to different functional outcomes, especially for weak antagonists. (C) Peptide motion relative to the V‐module during simulation (magenta). Viewing direction is from pMHC into the V‐module interface. The peptide from crystal structure is colored blue. In each panel, more than 10 snapshots from over 1‐μs long MD simulation are shown in semi‐transparent magenta, revealing degrees of motion. dFG: Cβ FG loop deletion mutant. The peptide angle is one of many measures that were used to quantify interfacial stability [61, 76, 86]. WT under high load (18.2 pN) best maintains the peptide position relative to the V‐module, as the peptide orientation stays closest to that of the crystal structure. (D) Positional shift of the MHC relative to the V‐module highlighted by the MHC α2 helix. For WT (“Tax”) under high load, the last frame from panel C (green and magenta for MHC α2 and peptide, respectively) is close to the crystal structure (brown and blue). Figure adapted from [61].

This contrasting αβTCR performance sensitivity directed at a single pMHC by two αβTCRs differing at one amino acid residue is analogous to the converse situation whereby a single αβTCR manifests differential responsiveness towards altered peptide ligands with only one residue difference relative to the cognate agonist peptide. A well‐documented case is that of the A6 TCR/Tax/HLA‐A*02:01 system in which the Tax peptide (11–19, LLFGYPVYV) derived from Human T‐cell leukemia virus type 1 (HTLV‐1) is recognized by an αβTCR isolated from T cells of patients with HTLV‐1 associated disease [12, 121]. A6 T cells showed differential responses when interrogated with variant peptides [121]. However, high‐resolution x‐ray crystal structures of these several variant complexes disclosed no significant structural changes that correlated with the biological effects [Figure 8A,B; [122, 123]]. Force‐dependent MD simulations (Figure 4E) revealed that the stability of the TCR‐pMHC interface depends on both the specific peptide and applied load through the dampening of the asymmetric interdomain motions by force and the organization and dynamics of interfacial contacts [Figures 5B and 8C,D; [61, 86]]. Main features of the motion of the TCRαβ chassis supporting the allostery, catch bond formation, and peptide discrimination were the same among three αβTCR‐pMHC systems tested, and are likely applicable across different αβTCRs in general [61, 76, 86]. For a viral or cancer epitope, immune evasion can be engendered via single site variants with minimal or no impact on biological function (for the virus or the tumor) but a significant impact on the protective immune response.

**FIGURE 9:**
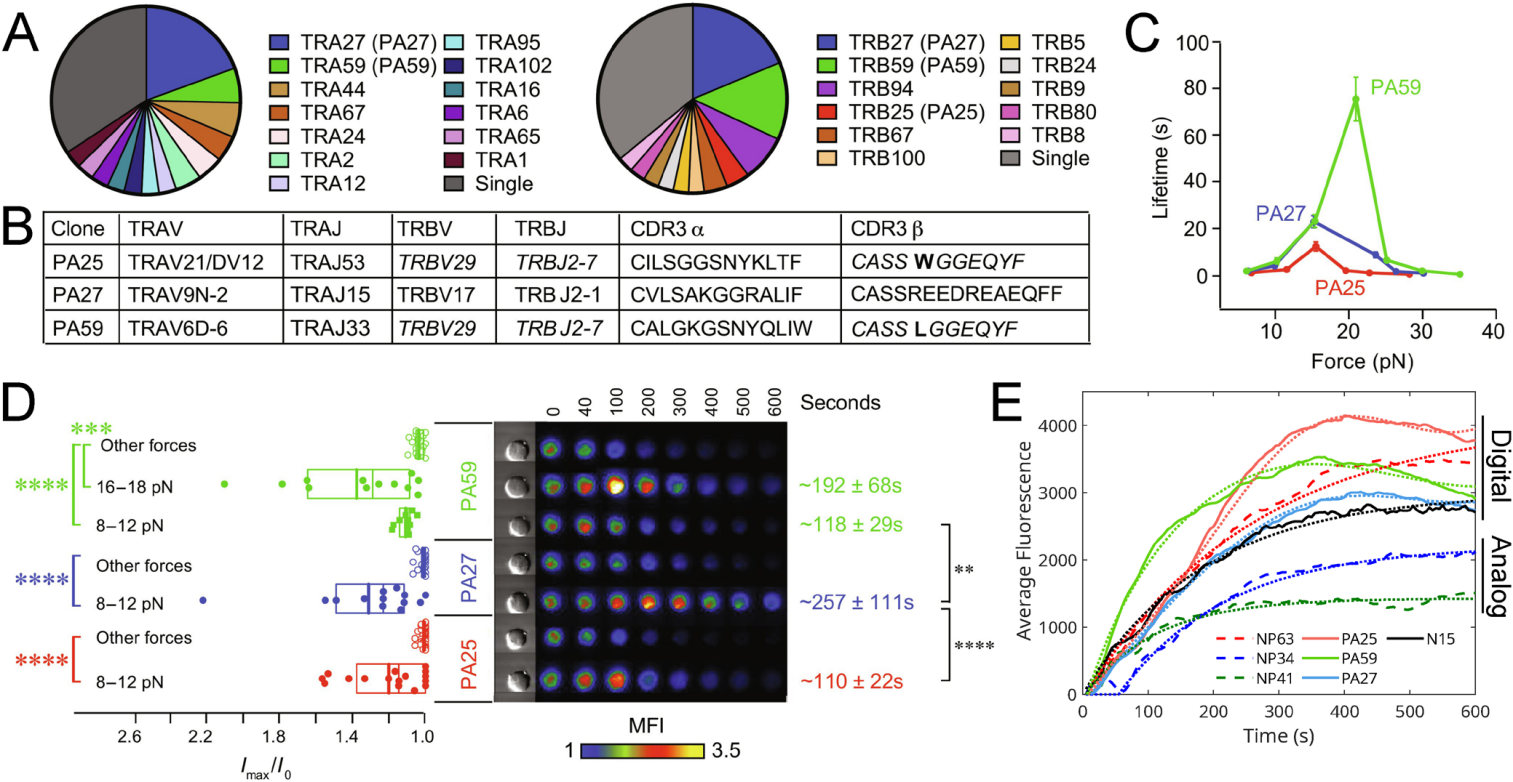
High sensitivity of TCRs specific for low‐copy ligand PA_224‐233_/D^b^ in mice challenged with IAV infection. (A) Proportions of V gene usage by PA_224‐233_/D^b^ specific T cells in recall responses to IAV. Note the top two TCRs (PA27 and PA59) account for a significant, but not as large, percentage of the repertoire as seen in the NP_366‐374_/D^b^ experiment (Figure 7A). (B) Gene usage for top three most common clonotypic TCRs as well as translation of CDR3 regions for TCRα and TCRβ. (C) SMSC Force‐lifetime plots of PA25, PA27 and PA59. (D) SCAR profiles of PA25, PA27 and PA59. Left: Relative maximum fluorescence intensity with triggering (I_max_/I_0_). Right: Representative triggered trace for each. Far Right: Average time to reach maximum fluorescence. Each TCR is shown at a copy number of 2 pMHC and at various forces and at optimal force needed to trigger. PA59 does not trigger at 8–12 pN but triggers well at 16–18 pN. (E) Metric for discrimination of digital versus analog TCRs by plotting average fluorescence versus time. Figure adapted from [60].

Responses to the sparser PA_224‐233_/D^b^ epitope following secondary IAV infection were also informative. Three of the top clonotypes were selected for biophysical characterization, which are PA27, PA59, and PA25, listed in the order of clonal abundance (Figure 9A,B). The SMSC force‐lifetime profiles show robust catch bonds with bond lifetime maxima greater than 10s (Figure 9C). Among them, PA59 exhibits a peak average lifetime of 80s with a force maximum at 20 pN, rather than 15 pN as observed in the other two. While each of these αβTCRs can be triggered at the digital threshold of 2 copies of pMHC in the SCAR assay, the PA59‐bearing T cells require a higher force for optimal triggering, concomitant with the higher force optimum determined by SMSC (Figure 9C,D). In fact, these most abundant clonotypes taken from the recall response for both NP_366‐374_/D^b^ and PA_224‐233_/D^b^ each exhibited differential sensitivity, including yet a third αβTCR characterized from the NP_366‐374_/D^b^ specific pool, NP41 (Figures 7 and 9D,E) which was weakly triggered and not digital in sensitivity. As more SCAR profiles are characterized, we can begin to build metrics for objectively assessing individual T‐cell clones, for instance by plotting the average Ca^2+^ fluorescence following triggering (Figure 9E). From this plot one can quickly assess the initial rate of Ca^2+^ release as well as maximal levels, where two subgroups of profiles emerge, corresponding to digital or analog responses. Standardization of this or other force‐activation frameworks will be critical in the future for correlating biological outcomes with the biophysical behaviors of individual αβTCRs. While the idea of an immunodominant response has been equated with successful response to an antigen, the diversity of T‐cell repertoires is not yet completely understood. If one can produce the digitally performing NP63, then are the less potent NP34 or NP41 a necessary part of the response? It is plausible that these suboptimal TCRs occupy valuable memory space as a hedge against viral evolution leading to altered epitopes against which these very TCRs manifest useful cross‐reactivities and hence immune protection. Alternatively, or in addition, it is because these T cells with less optimal TCR performance traffic to infected tissue more rapidly in the initial primary response than the more optimal TCRs [124]. For the question of PA27 versus PA59, one would suppose that individual differences would allow the different clonotypes to occupy separate niches in recognizing the same pMHC target. As discussed below, differences in tissue stiffness could impact the TCR‐pMHC interaction, meaning that optimal forces would vary with locale, particularly for resident memory cells.

### 3.2 αβT Cells and Ligand Scarcity

Another question regarding repertoires is the stringency necessary for an effective response when encountering a ligand presented at a given density. Cases of the IAV immunodominant ligands, specifically, NP_366‐374_/D^b^ and PA_224‐233_/D^b^, demonstrate the relevance of ligand density. Quantitative mass spectrometry analysis of IAV infected cells showed greater than 1000 copies per cell for NP_366‐374_/D^b^ while PA_224‐233_/D^b^, was present at less than 10 copies per cell [125]. One might predict that the need for digital response would be lesser in the case of the abundant ligand. While the number of αβTCRs we have interrogated is low, among the dominant clonotypes responding to PA_224‐233_/D^b^, all three have digital response capability, while among the top three responding to NP_366‐374_/D^b^, only NP63 was digital. In contrast, NP41 performed so poorly that obtaining binding data in the SM/SMSC context was challenging, likely due to a low on rate [60]. These results suggest that NP34 and NP41 are well suited as analog performers to their role in response to IAV because their ligand is so abundant. Apparently, nature selects performance to match the density of ligand array. Yet, one cannot assume that such is the case for a given ligand raised artificially through vaccination. If a digital performance is required, but high copy number of pMHC is arrayed through vaccination, there is a mismatch, assuming most vaccine‐engendered T cells are analog. Moreover, when assayed by standard IL‐2 response, the measure of which relies on bolus ligand dosing of APCs that yield unphysiologically high peptide density arrays, population‐level assessment of performance may not comport to in vivo protection outcomes.

### 3.3 Ligand Density and the Role of γδT Cells

**FIGURE 10:**
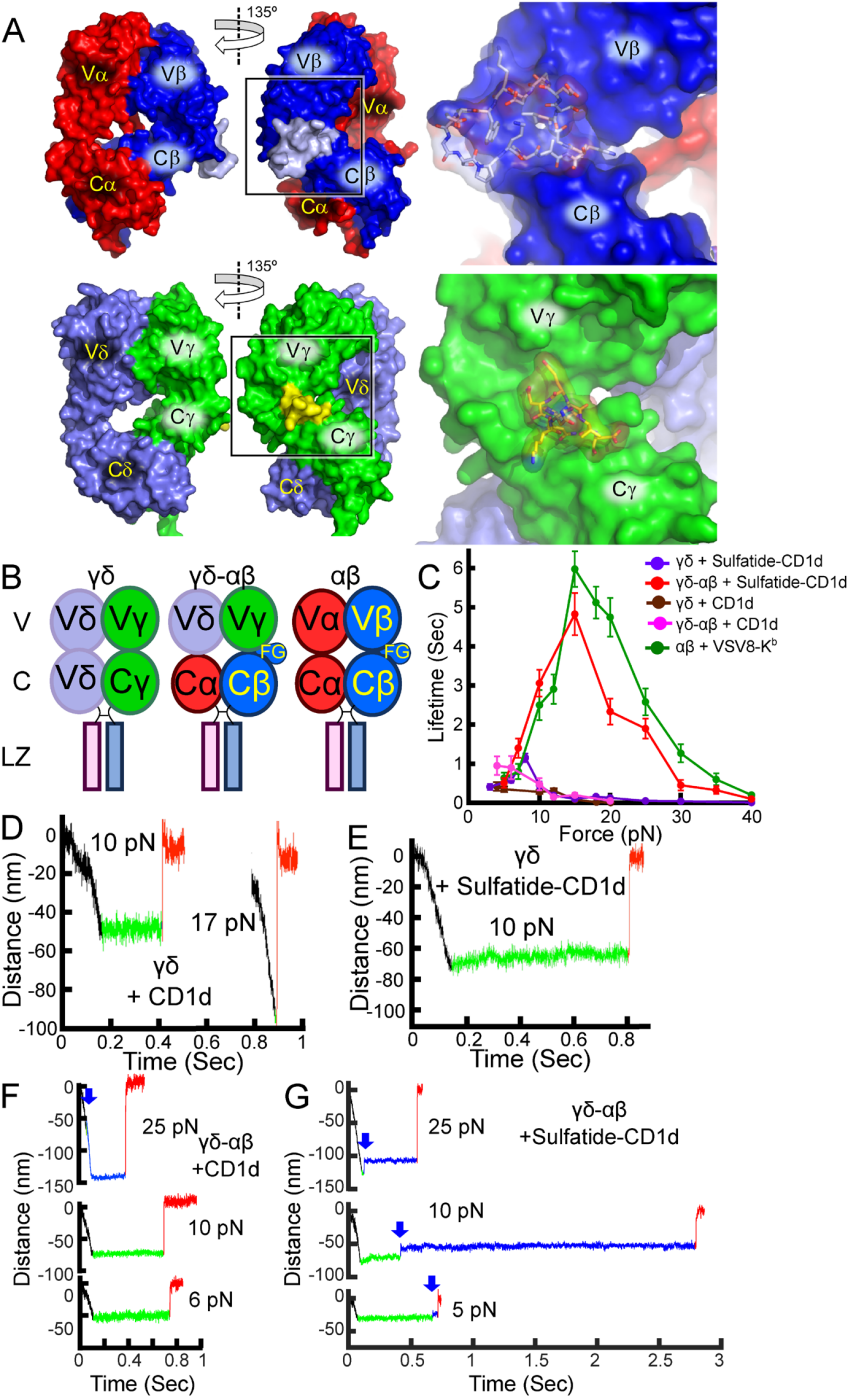
The γδTCR is not inherently force‐sensitive but can be engineered to be so by replacement of TCRγδ constant domains with those of TCRαβ. (A) Comparison between TCRαβ (top) and TCRγδ (bottom) ectodomains. Cβ FG loop is highlighted in light blue and Cγ FG loop is in yellow. Right column shows magnified views of the squared region. The FG loop has a broader area of interaction with the V domain in the TCRαβ compared to that of TCRγδ. (B) Construct design for SM experiments delineating allosteric mechanotransduction of the C domains of TCRαβ compared to that of DP10.7 TCRγδ. The leucine zipper (LZ) pairs allow interrogation of TCRγδ with CD1d‐sulfatide or CD1d (unloaded) in SM experiments. (C) SM bond lifetime vs. force curve of each construct with indicated ligand as compared to the N15αβ‐VSV8/K^b^ interaction. (D–G) SM traces of TCRγδ or γδ‐αβ chimera binding to CD1d or CD1d‐sulfatide. Each panel shows one or more single pulls with ligand to a specified distance (black trace), a pre‐transition dwell (green), if a transition is present, a post‐transition dwell (blue), and a rupture (red). Applied force is shown in each trace. Note that transitions are only common in experiments with chimeras and with CD1d‐sulfatide at most forces. Figure adapted from [36].

γδT cells are a diverse set of immune cells that occupy a range of physiologic niches with distinct antigen recognition capacity from the αβT cells, and clinical utilities [126, 127, 128, 129, 130, 131]. The recognition of a manifold set of ligands by the γδTCRs can occur in a variety of interaction modes, some like the canonical end‐to‐end recognition mode of αβTCRs and others recognizing side elements of the target molecule, which is generally a form of non‐classical MHC molecule [132]. The DP10.7 γδTCR recognizes sulfatide bound to the non‐classical MHC CD1d in a manner roughly analogous to that of TCRαβ‐pMHC interaction [133]. Structurally, the γδTCR complex resembles the αβTCR, utilizing the same CD3 components, but substitutes the evolutionary homolog TCRδ for TCRα and TCRγ for TCRβ (Figure 10A). This correspondence is significant given that the Vβ‐Cβ interface of TCRαβ is more robust [31] and the characteristically larger Cβ FG loop [36] has such profound effects on the mechanoreceptor properties of the αβTCR [31, 76]. In contrast, the TCRγδ heterodimer possesses a greater flexibility between the V‐ and C‐modules and additionally flexibility is imparted by its CPs which may enhance conformational flexibility in recognizing ligands in diverse orientations [134, 135].

We used SM interrogation of the γδTCR and a construct swapping the αβTCR C module for comparison [Figure 10B,C; [36]]. The TCRγδ could discriminate only weakly between unloaded CD1d and sulfatide‐CD1d, its preferred ligand [133, 136], with fast dissociation under force and without structural transitions Figure 10C–E; [36]. However, the γδ‐αβ chimera had significantly longer bond‐lifetimes under force with a characteristic catch bond and structural transition for the sulfatide‐CD1d, while for the unloaded CD1d, lifetimes were short and very few structural transitions were measured [Figure 10C,F,G; [36]]. This aligns with the principle that ligand binding and discrimination occur at the TCR‐pMHC interface via a combination of variable CDR1 and 2 with the hypervariable CDR3 loops.

While we emphasized the β‐γ comparison, Cδ is also considerably more structured than Cα, which may have an impact on the transitional propensity of the TCRαβ. The unusual structure of Cα has been the subject of speculation since the first structures of the TCRαβ were solved. Early researchers were cautious about the veracity of their structures until confirmation from multiple sources was obtained [12, 13, 14]. The contribution of Cα has not been thoroughly investigated, apart from the stabilizing effect observed with the addition of anti‐Cα H28 Fab on TCRαβ‐pMHC bond lifetimes [31, 137] and the previously mentioned preTCR experiments where the presence of Cα in TCRαβ compared to the preTCR had minimal impact on the critical force but slowed reversible transitioning in the former. Without the need for a readily deformable partner of Cβ, Cδ presumably confers more stability, similar to the homologous light chain of antibodies [138]. Importantly, the apparent uncoupling of V and C domains within TCRγδ relative to those in TCRαβ contributes to its flexibility observed in cryoEM [134].

For γδT cells, similar to the cases of αβT cells responsive to abundantly presented pMHC ligands, there appears to be no requirement for the highest sensitivity and strict force‐mediated ligand discrimination. Lipid antigens of γδT cells tend to be abundant [132, 139], and mechanisms by which recognition occurs include negative recognition wherein protruding self‐ligands inhibit binding until replaced by smaller permissive ligands [132]. In this case, digital recognition would not be advantageous, and may even lead to uncontrolled autoimmunity or exhaustion phenotypes. In the recognition of sulfatide‐CD1d by the DP10.7 γδTCR, γδ‐αβ chimeric T cells were more effectively activated by ligand‐loaded APCs during co‐culture experiments in a thymic stromal model system. This demonstrates the modular nature of the mechanotransduction apparatus even in TCRs separated by hundreds of millions of years of evolution [36, 134, 138]. The recognition of abundant ligands such as CD1a by αβT cells is unlikely to require digital sensitivity and these cells may behave more like γδT cells [139]. Similarly, canonical pMHC ligands in differential abundance would have differing activation requirements.

### 3.4 Mechanotransduction, Digital and Analog Initiation of Signaling

**FIGURE 11:**
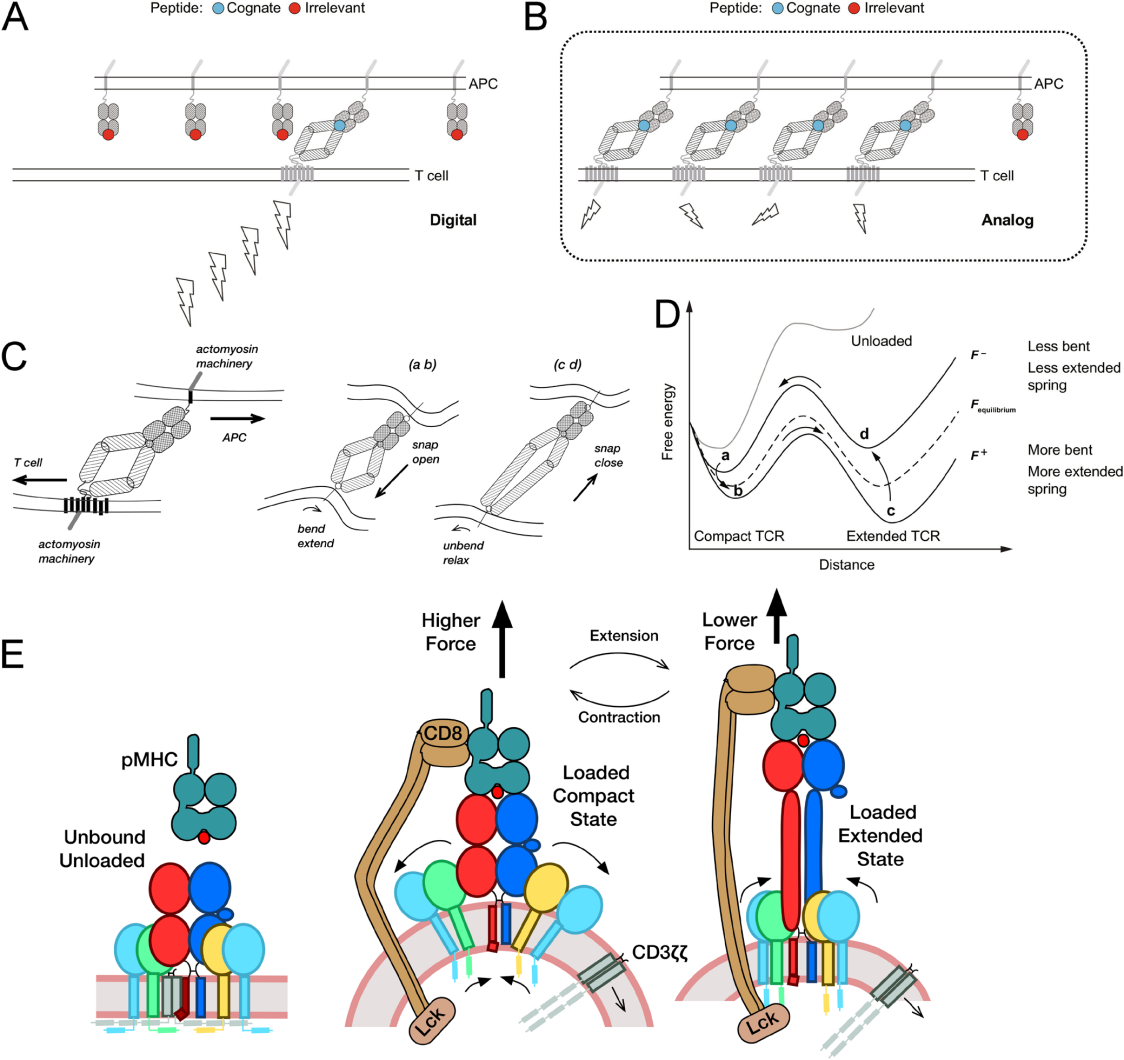
Models of TCR triggering and relationship with cytoskeletal elements. (A) Digital T cell triggering via sparse pMHC ligand results in highly energized TCR that transduces a strong signal despite low copy number. Signal initiation may be highly localized. (B) Analog T cell produces a weaker, more diffuse signal that relies on high copy number. (C) Linkage of TCR and pMHC to respective cell actomyosin machinery results in multiple cycles of opening and closing transitions generating local energy from cellular pools. Also see Figure 3C. (D) Dynamic oscillation of the free energy landscape. Transition to the extended state occurs when the applied load is high, causing rightward tilting of the free energy landscape. Once the extended state is reached, the larger span of the complex reduces force, resulting in leftward tilting of the free energy landscape. This leads to transition back to the compact state. With dissipative elements coupled to the TCR‐pMHC complex (e.g., membrane and the cytoskeleton; Figure 12), energy input is needed to maintain repeated cycling. Panels A‐D adapted from [58]. (E) Schematic of dynamic oscillation of the full αβTCR‐pMHC complex through repeated rounds of extension and contraction under load. This oscillation can agitate the membrane, re‐organize TM domains (e.g., dissociation of CD3ζζ), release ITAMs from the inner membrane leaflet, followed by their phosphorylation by Lck located on the cytoplasmic tail of CD8 (or CD4 on CD4+ T cells). Within the confined space between cells, pull on the membrane will be more during the compact (middle panel) and less during the extended phases (right panel) of the αβTCR‐pMHC complex. Transitions therefore bend the membrane as shown and agitate associations within the CD3 complex dimers. Similarly, since the conformational transition is thought to reside in Cαβ, local congestion due to the transient presence and absence of folded Cα and Cβ ectodomains will also agitate CD3 associations. Given that the CD8αβ coreceptor is positioned such that it extends from the membrane and interacts with the α3 domain of MHCI spanning the location of the conformational change, the extended state will yank on the CD8 tail driving Lck closer to ITAMs on the inside of the cell. Such positioning of the CD8 coreceptor can enhance signaling. The pMHC unbound state is in the left panel for completeness. Coloring of αβTCR is as in Figure 1A with CD8 in brown.

Figure 11 shows our current model of activation of the αβT cells with sparse and abundant ligands upon considering the experimental results discussed above. For sparse ligands, the T cell needs to find each copy of the target peptide among a field of unrelated self‐peptides (Figure 11A). This would correspond to the situation of the PA_224‐233_/D^b^ or certain cancer antigens of therapeutic interest such as the TP53 truncal neoantigen peptide R175H (^168^HMTEVVRHC^176^) bound to HLA‐A*02:01 [125, 142]. The second scenario occurs when the cell surface is populated with many copies of a target antigen relative to unrelated antigens (Figure 11B). This would be represented by NP_366‐374_/D^b^ [125], M1_58‐66_/HLA‐A*02:01 [143], or permissive ligands bound to CD1a [139]. The digital response is akin to the on/off state of single epitope recognition, a phenomenon long recognized [144] for T cells. The second analog case is termed in reference to the progressive increase in signal with each added ligand on the cell surface.

Figure 11C illustrates mechanotransduction in the digital response. Given the coupling of both the αβTCR and pMHC to the cytoskeletal framework directly or indirectly, the motion between a T cell and an APC provides a scanning mechanism [26, 32]. The pN‐nN forces inherent in this interface are much greater than forces exerted via Brownian motion. With surveillance‐facilitated access to the typical αβTCR optimal force for maximal bond lifetime and reversible structural transitions [31, 35], the TCR can cycle between compact and extended states without unbinding (Figures 3C and 11D).

The energetic drive for volleying comes from the actomyosin network (Figure 11C). Transitioning to the extended state accomplishes two things. First, due to the significant transition distance, if the “spring” that comprises the serial connection from the interaction interface inward to each of the cell membrane and cytoskeleton of both cells is properly matched, it will retract to accommodate this opening and force on the system will decrease, tilting the energy landscape leftward in the diagram, to favor a reverse transition to the compact state. This in turn would increase the applied load again and the energy landscape tilts to the right. Transition to the extended state follows, effectively restarting the cycle in a repetitive manner. The potential for this cycling is demonstrated in the dual trap SM experiments with the volleying behavior of digital TCRs [60]. This is not to suggest that in vivo the TCR‐pMHC bond would be controllable to the extent possible in the dual trap SM system, but it is plausible that evolutionary selection of the αβTCR tuned the system sufficiently to extend energized bond lifetimes for digital discrimination over many volleying cycles. The second aspect is the energy injection via volleying. This is a key aspect of differentiating the best αβTCRs from less optimal ones. If a single bond remains intact over the course of tens of seconds with a transition rate of tens of Hz, the free energy of several hundred transitions can be generated. This may agitate and energize protein–protein or protein‐lipid interfaces, inducing enhanced fluctuations in local membrane curvature, mechanical disruption of TCRαβ‐CD3 contacts and the lipids proximal to the αβTCR leading to reorganization of the TM region and unbinding of ITAM motifs from the inner leaflet of the membrane, exposing them for initiation of the phosphorylation cascade (Figure 11E). While details and sequence of the transition are currently not known, it is likely that multiple extensions occur simultaneously: the TCRαβ C domains reversibly unfold [31, 35, 118], connectors of low structural complexity such as the CPs may also be extended. Additionally, CD3 ectodomains are released from contacts with the TCRαβ ectodomains [17] and the outer leaflet of the membrane, and the TMs transition from a splayed to extended configuration [17]. Many of these changes could be coupled to phenomena described as phase separations [145, 146, 147] that have been linked to T‐cell function [148]. The repeated transitions could in theory promote exchange of the CD3 tails into phase separated clusters, a separation documented for T cells [148], for the LAT assembly [149], and to promote membrane receptor clustering through the actin cytoskeleton [150]. Clearly, transitions measured in SM using isolated ectodomains must be localized therein. Transitions have also been measured in SMSC interrogating the cell surface holocomplex even though they are more challenging experiments because the T‐cell surface tends to relax during bond measurement [31, 36]. SMSC experiments with TCRα TM mutants described above have also revealed complexities, such as the impact of the load propagation pathway on the signaling apparatus [63].

Analog signaling harnesses the same apparatus as digital recognition, but intercellular forces may be dispersed or otherwise hindered due to the greater ligand density. This may not be directly correlated with ligand density used in the SCAR assay. In the cellular context, forces may be dispersed across a parallel set of bonds that have different degrees of coupling to the same cellular machinery, rather than through a parallel set of interactions on a single optically trapped bead interfacing with the T‐cell machinery localized at the contact point. While a more nuanced view of the SCAR assay is thereby necessary, the accuracy of the assay is higher as one approaches the digital limit. Nevertheless, at higher ligand numbers, the αβTCR must utilize the same internal machinery to initiate and sustain cell signaling. Experimentally, in the absence of an external anchoring point, this is likely achieved by cross‐linked antibodies, such as the anti‐CD3 or anti‐TCR antibodies, or through streptavidin‐cross‐linked tetramer pMHC, binding tightly to the TCR components and forces actuated through the cytoskeletal machinery in an inside‐out manner terminating at the cross‐linked antibodies or pMHC, essentially pitting one TCR against the other. In an in vivo context, a stochastic array of instantaneous forces may act on a group of TCRs. For weaker activation, a subset of conformational changes detailed above integrated across a larger proportion of cell surface αβTCRs may achieve a threshold number for activation to start the signaling cascade. Analog αβTCRs can still undergo a single conformational transition, but their bonds do not survive repeat cycling. In the analog case the activation and ligand density would be directly related. This explains why bulk assays such as the IL2 secretion assay do not discriminate between analog and digital αβTCRs, as such assays test only the analog capacity of the αβTCR. A corollary is that digital αβTCRs can function perfectly well as analog αβTCRs, but not vice versa.

### 3.5 Adapting to Environmental Forces, Tissue Stiffness, and Compliance

**FIGURE 12:**
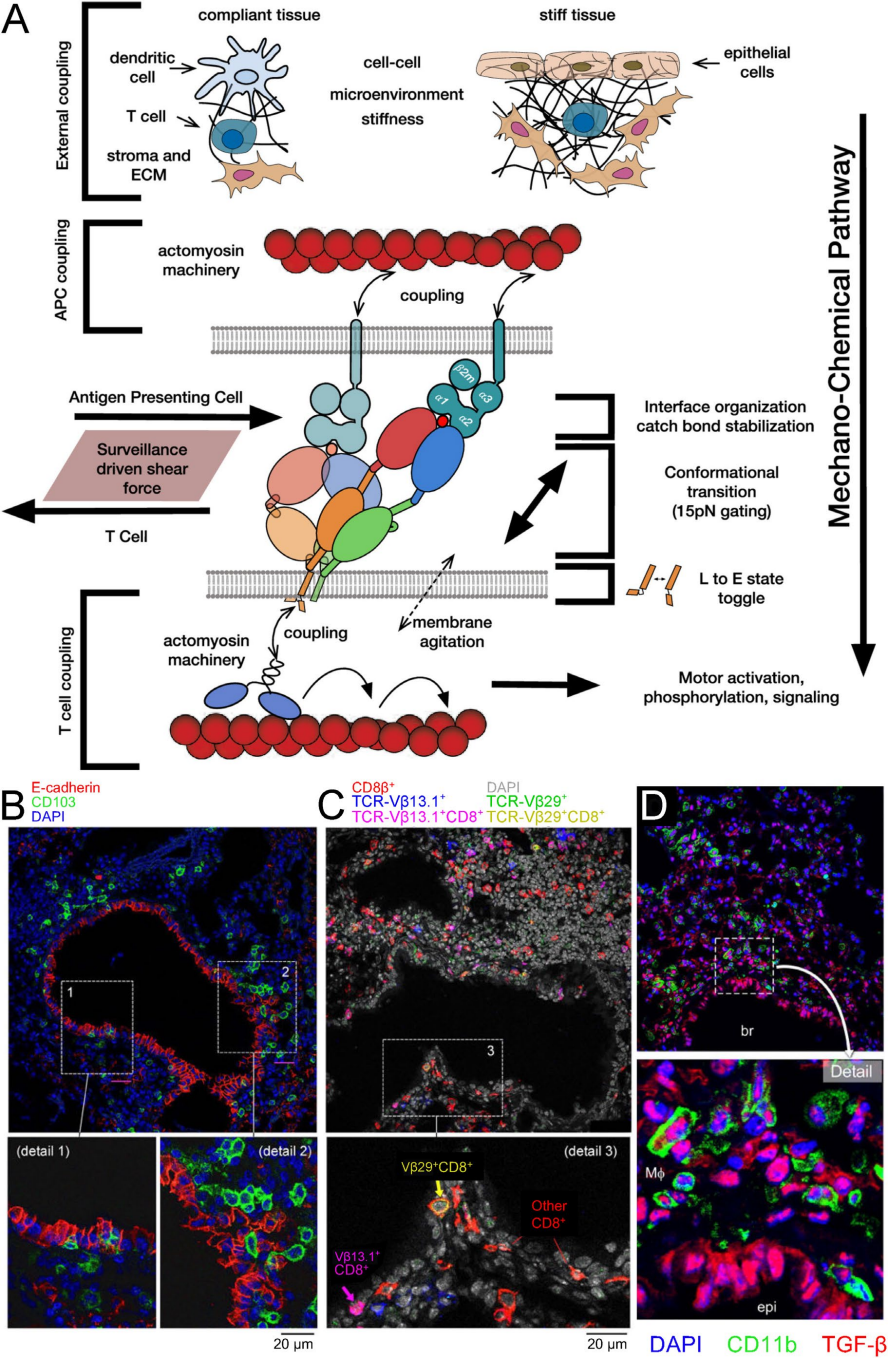
Mechanical properties of tissues have potential to impact mechanosensing. (A) Model of TCR mechanoregulation in tissues of varying stiffness showing forms of coupling, shear forces, conformational transitions and motor activation. Adapted from [58]. (B, C) Fluorescence microscopy of lungs isolated from IAV‐infected mice 7 days following recall response. (B) E‐cadherin stained cells (red) highlight the airway epithelial layer. CD103‐stained cells (green) show T cells and APCs. DAPI reagent (blue) stains nuclei. Insets at bottom show infiltration of epithelial cells with CD103^+^ cells. (C) CD8^+^ T cells (red) with Vβ13.1 [Vβ8.3^+^ = Vβ13.1 in modern nomenclature; [140]] cells (blue) and Vβ13.1^+^CD8^+^ cells (magenta), Vβ29 (TCRVβ7^+^ = Vβ29 in modern nomenclature) (green) and TCRVβ29^+^CD8^+^ cells (yellow). TRBV29 is the most common TRBV in recall responses to PA_224‐233_/D^b^ (Figure 9A,B) and TRBV13.1 is the most common TRBV in recall response to NP_366‐374_/D^b^ (Figure 7A,B). (D) Fluorescence microscopy of lungs isolated from IAV‐infected mice 14 days following primary infection. The lung was stained with anti‐TGFβ (red) and CD11b (green), the latter staining only macrophages (mΦ). Br = bronchioles. (B–D) adapted from [141].

Biophysical characteristics of a given αβTCR on a T cell are potentially matched to the target tissue undergoing surveillance, explicitly accounting for tissue stiffness generally, as well as within discrete anatomic sublocales. T cells infiltrate tissues to interrogate a variety of somatic cell types as well as professional APCs in situ. This suggests that the forces that the αβTCR experiences during immune surveillance will vary in magnitude, direction, and time‐dependence predicated on mechanical properties of its cellular and extracellular matrix milieu as illustrated in Figure 12A. Interaction between a T cell and an APC occurs while they are embedded within the extracellular matrix (external coupling). This involves various T cell to APC and cell to matrix adhesion molecules. Together with cytoskeletal organization occurring within both the T cell and APC (internal coupling), the TCR‐pMHC complex will experience a variety of forces.

As an example, the localization of CD8^+^ T cells in affected tissues was studied 7 days following secondary IAV infection using immunohistology and fluorescence microscopy [141]. Figure 12B shows the colocalization of CD103^+^ T cells and CD103^+^ dendritic cells (both green) with E‐cadherin^+^ epithelial cells (red), that line the bronchiolar space (black). A significant proportion of intraepithelial T cells as well as those in the inflammatory cell‐infiltrated subepithelial space (i.e., lamina propria) (Figure 12C, top) were identified with antibodies specific for TRBV13.1 and TRBV29 (Figure 12C, bottom detail). These are known to be dominantly utilized in responses to NP_366‐374_/D^b^ and PA_224‐233_/D^b^, respectively. The NP34 and NP63 T‐cell clones referenced above utilize TRBV13.1 (Figure 7A) while PA25 and PA59 both use TRBV29 (Figure 9A). While we cannot discern how the resident memory localization relates to mechanotransduction phenotype from this study, the clues are tantalizing in suggesting that TCR partitioning in tissue may vary based on clonotype, antigen specificity and individual biomechanical properties. Intraepithelial locale offers minimal compliance given the tight junction‐mediated connectivity of adjacent epithelial cells relative to the looser cellular packing of the subepithelial area. This latter region, however, can accommodate many inflammatory cells, as shown in the top of the field view in Figure 12C. Figure 12D further demonstrates that during primary infection both lung macrophages and epithelial cells generate TGFβ responsible for increasing extracellular matrix stiffness while diminishing tissue compliance. This leads to fibrotic responses persisting for weeks or even longer, and creates a challenging cellular milieu for the T cell to operate under force [reviewed in [58]]. The T‐cell repertoire for a given pMHC must accommodate such alterations through TCRs with differential bioforce parameters.

If one could determine which clonotype were localized in each locale, the relationship between mechanical force performance and tissue residence could be discerned using the methods described here. Moreover, the nature of cellular niches where individual T cells reside and which support the IAV response can also be defined. Technologies such as single cell spatial transcriptomics make these goals achievable [151].

For TCRs encountering a shift in cell–cell forces, the OT‐based assays can simulate different force‐regimes and system compliances by adjusting the spring constant of the trap. The function of each T‐cell clone within its memory niche is critical and likely varies along with cellular signaling components necessary for resident cell survival short and/or long term. Each of the αβTCRs of the IAV system discussed above (NP63, 34 and 41 and PA25, 27, and 59) are from T cells obtained at the recall response, being isolated several days post‐secondary viral challenge [60]. Further exploration of correlations between T‐cell function, optimum mechanical and niche cellular components is important to discern the richness of mechanobiology in cellular specification.

## 4 Translational Implications

### 4.1 Immuno‐Oncology: Adoptive T‐Cell Transfer and Cancer Vaccines

All considerations above impact approaches involving adoptive T‐cell transfer in cancer treatment and cancer vaccines. This has been discussed in detail recently and will be referenced briefly herein [58]. First, the quality of the TCR is paramount. If the target pMHC density is of sufficient magnitude on the cancer cell surface, an analog TCR may be sufficient. As with the NP_366‐374_/D^b^ antigens, while the recall response included abundant digital T cells, two of the top three clonotypes used were analog. On the other hand, with respect to sparsely expressed cancer antigens such as truncal neoantigens [22], the PA_224‐233_/D^b^ responsive repertoire is a more appropriate model. Due to the limitation on PA_224‐233_/D^b^ epitope abundance per APC, the T‐cell repertoire must be stringently sculpted by nature in order to mount an effective digital response. Clinically, achieving this goal is challenging particularly as it relates to vaccine elicitation, since counterintuitively, provision of high pMHC expression on APCs may yield unprotective analog responses. Adoptive T cell transfer will require digital performance as well. Hence, the procurement, elicitation, and testing of T cells should benefit from a sparse antigen challenge at every stage of evaluation.

Given the propensity for immune evasion, if the tumor tissue is not eliminated rapidly, an immunogenic epitope could be immune edited by the cancer to escape protective immunity over time [152]. Thus, it is imperative that any strategy includes a multi‐pronged approach. In light of the significant variability of T‐cell quality within a repertoire as demonstrated above, transfer of an antigen‐specific T‐cell expansion of an explicitly vetted, optimal digital performance is advisable. Furthermore, therapeutic utilization of T cells targeting at least several rather than single neoantigens and/or tumor associated antigens, although logistically demanding, is advisable. This strategy lowers the chances of immune evasion, particularly if each pMHC target utilizes a different MHC for presentation. Desmoplastic (i.e., fibroblastic tissue‐related) tumors will also create challenges for αβTCR function, akin to the considerations noted above involving non‐compliant tissue, unless force‐bond lifetime characteristics and biophysical parameters of αβTCRs are tuned appropriately to function in such an in vivo milieu.

### 4.2 Mechanisms of T‐Cell Signaling and CAR‐T Design

**FIGURE 13:**
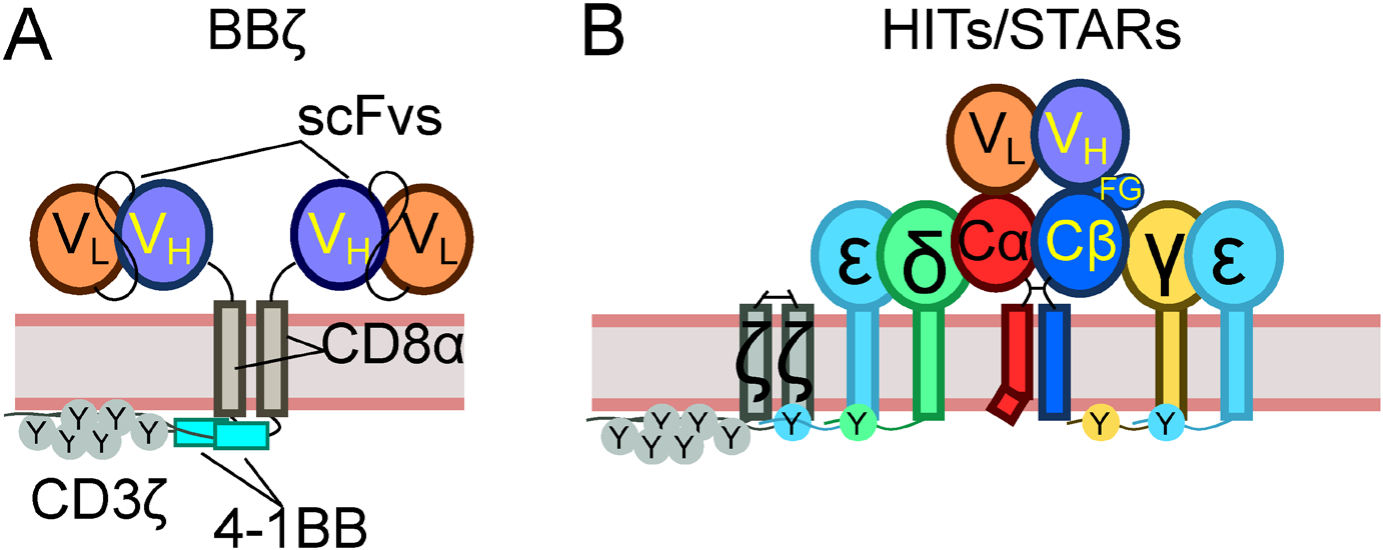
Design of therapeutics receptors (A). Commonly used BBζ CAR‐T receptor utilizing cancer‐specific scFV fragment fused to CD8α TM region, 4‐1BB cytoplasmic region, and CD3ζ ITAM region (B). Newer design of HITs or STARs, fusing light and heavy chain V regions individually with TCR C domains to form a TCR‐like CAR with potential for TCR‐like properties. Compare with domain organization of WT αβTCR (Figure 1A).

How, then, do chimeric antigen receptor T cells (CAR‐Ts) adhere to or violate principles of effective αβTCR function, and what can be done to engineer the most ideal specification? Figure 13A shows the design of a commonly used BBζ CAR‐T, consisting of paired single chain Fv regions of an anti‐cancer mAb fused each to the CD8α TM regions and linked to 4‐1BB costimulatory region and CD3ζ ITAM motifs in series. For abundant ligands like CD19, such a CAR‐T may function as an analog‐type T cell, relying on multiple weaker signals to effect antigen response on target cancer cells. This has been quite successful clinically in eliminating certain types of cancer, in particular leukemia and lymphoma, though escape is always a possibility through multiple mechanisms including downregulation of target antigen [153].

To replicate the regulation and sensitivity of the αβTCR (Figure 1A), researchers have fused cancer‐specific antibodies Fv domains with the C domains of TCRαβ, termed HLA‐independent TCRs (HITs), synthetic TCR and antigen receptors (STARs) (Figure 13B), costimulatory STARs (Co‐STARs), or MHC‐independent TCRs (miTCRs) [22, 154] which may be designed to be MHC dependent or independent. This approach has been successful in preclinical in vivo and in vitro studies with sensitivity thresholds approaching that of T cells responsive to R175H/HLA‐A*02:01 [22]. Paramount in understanding the efficacy of this approach is defining the force‐response capability of the chimeric HIT in driving downstream signaling. Specifically, how well do the novel interfaces between the V and C domains replicate that found in native αβTCRs? Are these HITs digital or simply high‐affinity analog? If the latter, how is it possible to respond to the low copy‐number R175H/HLA‐A*02:01 epitope, which was reported to be presented at 1–2 copy/cell [22]? Do such approaches work in vivo as chimeric receptors or as digital αβTCRs?

## 5 Concluding Remarks

The mechanosensing paradigm provides guidance in addressing both infectious pathogen‐derived targets as well as cancer epitopes. Understanding the underlying biology of the disease target is essential, particularly regarding the copy number of target epitopes displayed on the APC surface. This can be accomplished through genetic and transcriptomic analysis to identify expressed targets, especially in the case of cancer and mutated proteins in conjunction with quantitative mass spectrometry to define and quantify the actual cell‐surface presentation of such epitopes in the immune peptidome of arrayed pMHCs. For infectious diseases, the biology of each pathogen will dictate the cellular and temporal distributions of protein expression, in turn subject to stereotypical proteosomal degradation, TAP transport to the endoplasmic reticulum, peptide loading apparatus‐mediated insertion into MHC molecules, and transport and display on the cell surface. Deeper understanding of these processes will allow one to evaluate the T‐cell pool against immunoprotective epitopes for digital or analog clones as appropriate for vaccine design and elicitation or TCR selection for adoptive therapies. In this manner, αβTCR mechanobiology can be leveraged broadly for effective clinical applications. Since the evolutionary selection of the αβTCR has integrated force for optimal function in protecting organisms from disease, therapy reliant on T cells must also do so through detailed and nuanced understanding of these mechanosensing principles.

## Conflicts of Interest

The authors declare no conflicts of interest.

## Supporting information


**Video S1.** T cell scanning an epithelial cell surface in search of pMHC. Movie reveals motion of T cell scanning cellular substratum from which serial stills in Figure 3A are extracted. Movie is from Tibaldi, Salgia, and Reinherz [57].

**Video S2.** T cell scanning an epithelial cell surface in search of pMHC. Movie reveals a T‐cell leading edge scanning cellular substratum while a uropod remains fixed. Movie is from Tibaldi, Salgia, and Reinherz [57].

## Data Availability

Data sharing not applicable‐no new data generated.
